# Micro- and nano-devices for electrochemical sensing

**DOI:** 10.1007/s00604-022-05548-3

**Published:** 2022-11-22

**Authors:** Federica Mariani, Isacco Gualandi, Wolfgang Schuhmann, Erika Scavetta

**Affiliations:** 1 Dipartimento Di Chimica Industriale “Toso Montanari”, Università Di Bologna, Viale del Risorgimento 4, 40136 Bologna, Italy; 2 Analytical Chemistry - Center for Electrochemical Sciences (CES), Faculty of Chemistry and Biochemistry, Ruhr University Bochum, Universitätsstraße 150, 44780 Bochum, Germany

**Keywords:** Electrochemical sensor, Microelectrode, Nanoelectrode, Transistor, Miniaturization

## Abstract

Electrode miniaturization has profoundly revolutionized the field of electrochemical sensing, opening up unprecedented opportunities for probing biological events with a high spatial and temporal resolution, integrating electrochemical systems with microfluidics, and designing arrays for multiplexed sensing. Several technological issues posed by the desire for downsizing have been addressed so far, leading to micrometric and nanometric sensing systems with different degrees of maturity. However, there is still an endless margin for researchers to improve current strategies and cope with demanding sensing fields, such as lab-on-a-chip devices and multi-array sensors, brain chemistry, and cell monitoring. In this review, we present current trends in the design of micro-/nano-electrochemical sensors and cutting-edge applications reported in the last 10 years. Micro- and nanosensors are divided into four categories depending on the transduction mechanism, e.g., amperometric, impedimetric, potentiometric, and transistor-based, to best guide the reader through the different detection strategies and highlight major advancements as well as still unaddressed demands in electrochemical sensing.

**Graphical Abstract:**

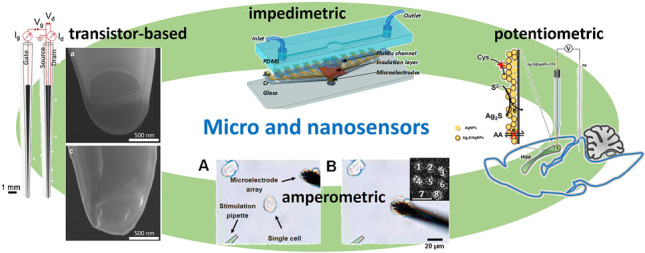

## Introduction

Although the first electrochemical sensor appeared in the first decade of the twentieth century [1], the number of biosensor-related publications experienced a sharp increase only after the pioneering works of Clark [2], who published his O_2_ electrode in 1956 [3]. A few years later, he reported the first example of electrode systems for continuous monitoring of blood composition [4], including the description of an enzyme-based electrochemical sensor for glucose detection. Interestingly, the first experiment involving the fabrication of Au ultramicroelectrodes (UMEs) came far earlier in 1801 [5], while a microburner-assisted method for the fabrication of a glass micropipette for the manipulation of single bacteria was reported one century later [6] and can be considered as the ancestor of current pulling methods. Shortly after, in 1921, the first successful attempt in the production and use of a microelectrode for the electrical stimulation of living cells was made by Hyde [7, 8]. Since then, UMEs have remained a niche class of electrochemical tools for decades, mainly due to technological limitations in recording the especially small currents associated with their unique size-dependent features. In the 1980s, the renewed interest in the in vivo recordings of neurotransmitters, as well as in the scanning and interrogation of surfaces, culminated with the first microvoltammetric electrodes for dopamine detection in brain tissue [9] and the invention of scanning tunneling microscopy (STM) [10, 11] and scanning electrochemical microscopy (SECM) [12], respectively [13]. These events represent a milestone in the development of UMEs to access unique electrochemistry applications, which have more recently benefited from the advancements in micro-/nanoscale fabrication and machining technologies. In the same years, starting from the late 1950s, the complementary metal–oxide–semiconductor (CMOS) technology has been revolutionizing the whole electronic manufacturing industry leading to the massive spreading of microchips, integrated circuits, and transistors.

By definition, UMEs have at least one dimension, e.g., the critical dimension, such as the radius of a disk electrode, smaller than 25 µm, thus falling into the scale of typical diffusion layer thicknesses [14]. However, we feel that a more appropriate definition is an electrode with at least one dimension, for which in the timescale of the chosen electrochemical experiment a stationary diffusion layer is reached. This includes that, e.g., at fast voltammetric scan rates even for small electrode dimensions a typical Cottrell-type planar diffusion is observed, which makes this electrode in combination with the speed of the voltammetric scan behave as macroelectrode. In the case of nanoelectrodes, their size in at least two dimensions is substantially below 1 μm [15]. The lower limit in downscaling the critical dimension is typically set to 10 nm, where the electrode size approaches the thickness of the double layer or molecule dimensions and originates a peculiar electrochemical behavior that deviates from theory [14, 16]. The small size of micro- and nanometric probes is the basement for their peculiar electrochemical features, including (i) reduced RC constant (cell time constant), which greatly influences the time resolution and allows measurements of fast processes; (ii) reduced iR drop, thus allowing measurements with simplified instrumentation (from three to two-electrode setup) in scarcely conducting media, such as non-polar solvents or real matrices, and in the absence of supporting electrolyte; (iii) improved signal to noise ratio (enhanced Faradaic over non-Faradaic current ratio) and increased diffusional mass transport rate; (iv) relative insensitivity to convection thanks to the high mass transport rates, thus allowing in-flow electrochemical measurements; and (v) high spatial resolution and minimal invasiveness, thus causing negligible system perturbation [14, 17–20]. Such unique features of UMEs have opened the way to a broad set of otherwise inaccessible applications, ranging from the fundamental understanding of fast redox process kinetics to quantitative analysis of brain chemistry.

For the correct interpretation of the electrochemical information obtained from the micro-/nano-sized probe, its geometry must be defined allowing to model electrode processes and mass transport phenomena. In this regard, the fabrication process plays a key role. Micro-/nanometric sensors can be roughly divided into two categories, i.e., chip-like and pipette-like, depending on their macroscopic shape that will be either planar and flat, or vertical and sharp, respectively. For historical reasons, conventional electrochemistry with micro- and nanoelectrodes mainly relates to pipette-like probes providing a high aspect ratio and tunable disk-shaped of the hemispherical-shaped geometry and allowing precise positioning with high spatial resolution by the exploitation of scanning electrochemical microscopy setups. This kind of miniaturized probe is mainly obtained by encapsulating or sealing the electrode material, such as carbon fibers or metal wires, in an insulator like pulled glass/quartz capillaries or polymer bodies. Thereafter, the electrode tip is exposed by mechanically polishing or chemically etching of the insulator. Technical aspects and recent advancements in carbon nanoelectrode fabrication, including the flame-etching of carbon microfibers, chemical vapor deposition (CVD) of a carbonaceous layer inside of pulled quartz capillaries with methane gas as a precursor, and pyrolysis of a propane/butane gas mixture have extensively been reviewed elsewhere [15, 16, 21]. On the other hand, the chip-like miniaturized structure has evidently benefited from the rapid evolution of microelectronics and the integrated circuit industry and relies on thin-/thick-film techniques employed in bottom-up manufacturing technologies including physical vapor deposition, photolithography, electron-beam lithography, and focused ion beam (FIB) milling. On the one hand, these approaches are more prone to automatization and large-scale production, while on the other hand, they require expensive facilities that impact the cost of the final device [22–25].

In this review, we will focus on the major advancements achieved during the last decade in the development of micro- and nanoelectrodes for sensing applications in analytical chemistry. The contributions discussed in this review are organized according to the transduction mechanism originating from the sensing process occurring at the micro-/nanoprobe, e.g., amperometric, potentiometric, impedimetric, and transistor-based.

## Micro- and nano-sized amperometric sensors

In controlled potential methods, potential steps or sweeps are applied to the working electrode. As a consequence of this perturbation, electrochemical reactions involving the analyte may occur at the interface, where the resulting current is monitored and can be correlated with the analyte’s concentration. Downsizing of the electrode dimension leads to improved mass transport and resolution of the electrochemical experiment. Moreover, miniaturization of the probes facilitates the design of arrays, interdigitated structures, and integration with microfluidics and implantable devices. Furthermore, nanoscale effects leading to a higher electric field near the electrode promote electron tunneling and further influence the mass transport of electroactive species, thus improving electrical communication with the redox center of enzymes, achieving high sensitivities in biosensing [26] and allowing high-frequency recordings. For these reasons, micro- and nano-sized amperometric sensing devices have mainly been applied to neurotransmitters and neural signal detection, DNA and protein detection, bacteria detection, cell metabolism monitoring, and detection of biomarkers in biological fluids.

### Neurotransmitters and neural signal detection

**Fig. 1:**
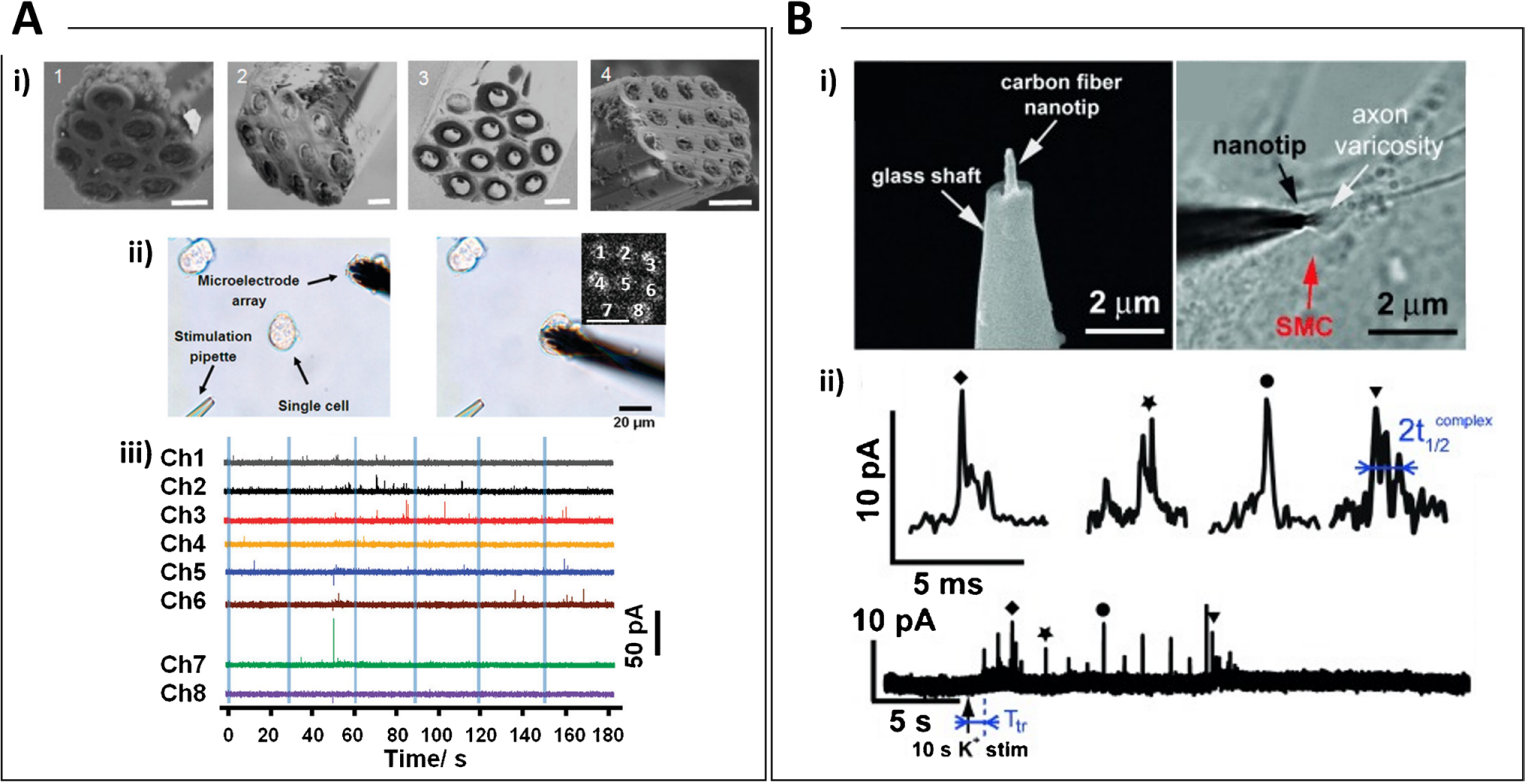
**A** Carbon-ring microelectrode arrays (CRMAs) for electrochemical imaging of single cell exocytosis. i) Scanning electron microscopy of CRMAs having (1) eight, (2) ten, (3) twelve, and (4) fifteen microring electrodes. Scale bars 5 μm. ii) Optical images showing an eight-electrode CRMA before (left) and after (right) positioning on a single PC12 cell. Inset: electrogenerated chemiluminescence image of the used CRMA (scale bar: 10 μm). iii) Amperometric traces of exocytotic release from a PC12 cell recorded using an eight-electrode CRMA. Blue stripes indicate high potassium stimuli. Reprinted with permission from [38]. Copyright 2012 American Chemical Society. **B** Real-time monitoring of discrete synaptic release events within self-reconstructed neuromuscular junctions. i) (left) SEM of a carbon fiber nanoelectrode and (right) bright-field optical micrographs showing the tip of a sensor inserted inside a synapse between a varicosity of a superior cervical ganglion (SCG) neuron and a smooth muscle cell (SMC). ii) High K^+^-induced amperometric spikes with the four labelled typical complex events being enlarged above. Reprinted with permission from [52]. Copyright 2015 WILEY–VCH

Neuroscience deals with the complex mechanisms of synaptic transmission. Real-time monitoring of neurotransmitters signaling pathways and anomalies is essential to understanding the etiology and progress of neurodegenerative diseases. Despite only ascorbate was detectable, the feasibility of neuroelectrochemistry experiments in brain tissue was demonstrated by Kissinger et al. in the early 1970s [27], when a carbon paste–based microelectrode was used to carry out in-vivo voltammetry and, in contrast to microdialysis-based methods, shed light on the opportunity to monitor neurotransmission fluctuations in real-time. Since then, starting with the works by Wightman’s group with detection of neurotransmitters both in vivo [28, 29] and adjacent to single cells in vitro [30, 31], carbon fiber (CF) microelectrodes have dominated the biological field. Thanks to their biocompatibility and dimension, typically below 10 µm, CF microelectrodes are suitable for implantation and non-toxic to living cells, while a Nafion coating is typically applied to block the interference from negatively charged molecules such as ascorbate [28]. Moreover, thanks to the relatively constant charging current allowing facile background subtraction, CF are especially interesting for fast-scan cyclic voltammetry (FSCV) measurements, which offer higher selectivity than high-speed chronoamperometry [32] and higher signal-to-noise ratio if compared to constant-potential amperometry [28]. The measurement of exocytotic dopamine (DA) secretion from individual pheochromocytoma (PC12) cells, a model cell line for neurosecretion, has been carried out by the Zhang group using Au-functionalized CF microelectrodes [33, 34]. Single-cell amperometry was performed by gently lowering the micro-sized probe toward a cell of interest, demonstrating improved electron-transfer kinetics and electrocatalysis at the CF, thanks to the presence of a Au film electrodeposited through a voltage-pulse method [34]. In vivo FSCV was carried out upon insertion of CF microelectrodes in the striatum of primates, where DA variations during reward delivery were found to be masked by pH changes and increases in extracellular O_2_ [35]. Due to the typically small basal DA concentration (low nM range) detectable in vivo, the presence of abundant electroactive interferents and the numerous complications related to implantation [36, 37], plenty of studies have been reported dealing with extracellular somatodendritic or axonal DA release from brain slices, as well as detection from cultured neurons. In this regard, the use of microelectrode arrays can provide information about the spatial variability and distribution of neurotransmitter release events. A microring electrode array (Fig. 1A) containing up to 15 individually addressable carbon electrodes was reported to spatially monitor exocytotic release at PC12 cells by chronoamperometry [38]. Multiwalled carbon nanotubes (MWCNTs) electroplated planar indium tin oxide (ITO) multielectrode arrays (MEAs) [39] and Au MEAs [40] were developed for the detection of DA in striatal slices and cultured neurons (also combined with electrophysiology measurements) and at four structural locations of isolated dopaminergic somas from the pond snail, respectively. Amperometric measurement of the quantal DA release of neuronal synaptic vesicles as well as spontaneous neuronal firing activity in vitro was achieved with a micro-graphitic single crystal diamond based MEA [37]. Under physiological conditions (2 mM Ca^2+^), the microarray probe was able to resolve spontaneous secretory events as amperometric spikes of < 20 pA I_max_ with half-time width of 0.57 ms.

Further downsizing of the amperometric probe dimension to the nanometre scale improves the resolution of the measurement and can enhance the analytical performance of the sensing device. Detection of DA in the presence of ascorbic acid (AA) was studied using linear sweep voltammetry (LSV) with wafer-scale manufactured Au nanoelectrodes having 30–500 nm width [41] and using redox cycling with 150 nm width Au interdigitated electrodes (IDEs) [42], demonstrating improved analytical performances with respect to standard Au thin-film electrodes and faradaic current enhancement, respectively. An implantable 800-nm diameter Au nanotip modified with Au nanoclusters and Nafion was employed as amperometric DA sensor in the striatum of rats, where the concentration of evoked DA release was estimated to be 37 nM [43]. The analytical performance of a carbon nanopipette electrode (CNPE) with ∼250 nm tip diameter and controllable length of exposed carbon, ranging from 5 to 175 μm, was characterized for neurotransmitters detection by FSCV, demonstrating higher sensitivity in serotonin sensing than traditional CF microelectrodes [44]. Thanks to the small dimension, the nanotip was used to monitor DA release at the dopaminergic centers of the fruit fly brain. Very recently, the first example of a 3D-printing approach applied to free-standing nanoelectrodes was reported [45]. After direct laser writing onto metal wires, the polymerized photoresist was pyrolyzed providing a glassy-carbon-like surface, then insulated by atomic layer deposition of Al_2_O_3_ and polished to disk-shaped with 600 nm diameter by a focused ion beam. The small size allowed insertion in adult fruit fly brain, where stimulated release of DA was detected by FSCV.

For the detection of non-electroactive neurotransmitters, the most common strategy involves the immobilization of oxidoreductases at the electrode surface. The locally generated H_2_O_2_ is then electrooxidized at the microbiosensor and its concentration can be determined from the current generated during a simple constant-potential amperometric measurement. For instance, glutamate concentration was measured in human embryonic stem cell–derived organoids, e.g., 3D cell models that are considered to better mimic the complexity of brain architecture and functioning with respect to conventional flat cultures. Glutamate oxidase (GluOx) was immobilized on the Pt micropipette electrode using bovine serum albumin (BSA) as the stabilizer, preserving the 3D network of the enzyme, and glutaraldehyde (GA) as the cross-linker, while an electrodeposited film of poly(m-phenylenediamine) (PPD) served as a permselective membrane [46]. An analogous immobilization approach was employed with glucose oxidase (GOx) to fabricate a platinized CF microbiosensor for the detection of glucose fluctuations in hippocampal brain slices in response to depolarization events [47]. In another recent report, glutamate was detected using a Pt nanoparticle (NP)/GluOx/CF microbiosensor during exocytosis from single hippocampal neurons [48]. In this case, the enzyme was immobilized at the electrode surface using the polycationic stabilization agent polyethyleneimine (PEI) and poly(ethylene glycol) diglycidyl ether (PEGDE) as the cross-linker, the latter often reported as superior to GA due to the absence of GA handling hazards and the lower impact on the response time of the device [49].

At the synaptic cleft, e.g., the narrow gap of a few tens of nanometer separating two neurons, neurotransmitters transients in the millisecond timescale occur, with concentration peaks reaching the mM range that rapidly decays due to diffusion, re-uptake, binding to receptors/transporters, or enzymatic breakdown [50]. Only advances in nanoelectrode fabrication have facilitated the manufacturing of sufficiently small sensing probes to overcome such limited accessibility in space and time for quantitative experiments, which have been recently reviewed by Shin et al. [51]. Real-time monitoring of discrete vesicular release of neurotransmitters inside neuromuscular synapses and excitatory potentials recordings were first demonstrated with a microfluidic device by Li et al. [52], where a flame-etched conical carbon nanofiber electrode and a glass nanopipette electrode served for amperometric and action potential measurements, respectively (Fig. 1B). More recently, in situ simultaneous measurement of synaptic acetylcholine (ACh) concentration and the release dynamics was reported with a nanoelectrode of ∼15 nm in radius, which was positioned at the synaptic cleft using nano-resolved SECM [53]. The selective amperometric detection of ACh exploited the charge transfer across a nanointerface between two immiscible electrolyte solutions (ITIES) supported on the orifice of a nanopipette. This strategy based on nanoITIES for the detection of non-electroactive neurotransmitters was demonstrated also for γ-aminobutyric acid (GABA) exploiting pH modulation from the oil phase [54].

### Biological markers detection

The pH value is an essential parameter influencing thermodynamics and kinetics of most homogeneous and heterogeneous processes, and its electroanalytical determination at the microscale is usually performed with potentiometric probes, which however suffer from the limited accuracy in open circuit potential (OCP) measurements caused by miniaturization. A voltammetric microsensor was realized upon oxidation of a CF surface to exploit the quinone/hydroquinone redox couple for pH sensing [55]. O_2_ interference was removed using high scan rates, and the sensor was validated in human saliva. Functionalization of carbon nanoelectrodes (CNEs) with the pre-adsorbed water-insoluble compound syringaldazine was reported by Michalak et al. [56]. Thanks to the 50 nm diameter of the nanovoltammetric probe, the authors were able to map pH variations over a Pt UME during ORR.

Amperometric detection of glucose continues to attract considerable attention due to its key role in biochemistry, healthcare, and food analysis. Examples of enzymatic micro- and nano-sized biosensors include Pt microelectrode–supported hydrogels modified with electrodeposited polypyrrole (PPy) in the presence of GOx [57] and carbon interdigitated nanoelectrodes detecting glucose in human serum by redox cycling in the presence of a redox mediator [58]. Moreover, a highly integrated Au MEA biosensing platform was realized for the detection of glucose, lactose, phenolic compounds, and herbicides for agrifood screening [59]. Another relevant example of an integrated biosensing platform was given by Hoa et al., with the design of a micromachined probe composed of a micro-Pt working electrode, a micro-Ag/AgCl reference electrode (RE), and a micro-Pt counter electrode (CE) [60]. In this case, glutamate detection was carried out in human serum upon functionalization of the sensing area with GluOx and an over-oxidized PPy/Nafion permselective membrane. The same functionalization strategy was employed to build an amperometric Pt microbiosensor for the detection of alanine aminotransferase (ALT), a clinical biomarker for hepatic dysfunctions, in human serum exploiting an enzymatic reaction cascade yielding H_2_O_2_, eventually directly oxidized at the microbiosensor surface [61]. Concerning non-enzymatic sensing in alkaline solution, Jiang et al. fabricated a Cu NP–functionalized linear graphene edge nanoelectrode with a thickness of 1.7 nm, which was tested for glucose detection within the medically relevant concentrations in alkalinized blood samples [62].

Hydrogen peroxide is an important biomarker of oxidative stress, and its detection is essential for the majority of oxidase-based biosensors. Examples of amperometric H_2_O_2_ sensors were reported using nano-sized Ag particles [63] and Pt NP [64]–modified CF microelectrodes, both of them employing Nafion and/or PPD membranes to achieve the desired selectivity. In the first case, H_2_O_2_ decomposition over MnO_2_ catalyst was monitored in real time exploiting its reduction at 0 mV vs Ag/AgCl, including high decomposition rates conditions that are typically inaccessible for titrimetric, chromatographic, or spectroscopic methods [63]. In the second example, Wang et al. proposed a fast metallization method to cover the CF microelectrode with Pt nanostructures and demonstrated good selectivity to H_2_O_2_ despite the oxidative potential employed during the detection (+ 0.7 V vs Ag/AgCl) [64].

A key advantage of miniaturized amperometric sensors is the facile integration with a microfluidic platform, allowing to miniaturize the sample volume and to simplify handling due to the elimination of mechanical convection. These aspects are particularly convenient when analyzing reactive molecules and free radicals, such as NO, characterized by a fleeting existence and extremely low concentrations in biological fluids [65]. To this regard, a microfluidic, xerogel-modified Pt microelectrode was realized allowing selective amperometric detection of NO in simulated wound fluid and whole blood for monitoring inflammatory responses to infections [66]. Due to the reduced noise level caused by the microfluidic configuration, a detection limit of ~ 500 nM was obtained in blood, which is unfortunately still too high for physiological NO concentrations (nM range), but compatible with the monitoring of NO-related diseases (µM range).

Another important biomarker for immune response monitoring is interleukin-6 (IL-6). A needle-shaped array of photolithographically fabricated Au microdisk electrodes was recently reported for IL-6 immunosensing, exploiting the formation of a self-assembled monolayer for antibody binding. The detection was carried out by differential pulse voltammetry (DPV) at clinically relevant concentrations (pg/mL) [67]. Other examples of micro- and nano-sized immunobiosensors have been reported for the detection of prostate-specific antigen (PSA) as a prostate carcinoma biomarker [26] and methylated DNA as a target for early tumor diagnosis and monitoring [68]. A microfluidic biosensor chip including Ag/AgCl and Au electrodes as RE and CE, respectively, and a Au nanoelectrodes array functionalized with a biosensor complex enabled the voltammetric determination of PSA with fM detection limit in nL volumes using a competitive immunoassay method [26]. A PPy/Pt microdisk MEA was realized for the voltammetric detection of DNA methylation in a concentration range between 0.001 and 0.1 μM [68]. This strategy exploits the hindered transport of Cl^−^ ions due to methylated DNA forming a compact duplex with the ssDNA probe, which is attached to the anion exchanging PPy transducer where ion-to-electron conversion occurs. Furthermore, the use of nanostructured Au microelectrodes (NME) in combination with electrochemical steric hindrance hybridization assay was described for the detection of a model antibody, demonstrating improved analytical performances compared to macroelectrodes due to size-dependent hybridization rates and morphology-induced blocking effects [69].

Another interesting approach employs the single-particle collision method, where the stochastic collision of single particles blocks the flux of redox species undergoing oxidation or reduction at a UME. Bacteria [70] and human platelets [71] have been detected using CF and Pt UMEs, respectively. In the first case, the positive electric field originating from the steady-state current oxidation of ferrocyanide stimulated the migration of negatively charged *E. coli* bacteria towards the UME, eventually hindering access to the probe surface and causing a decrease in the recorded current. In the second one, the concentration of ferrocyanide in solution was optimized to facilitate the migration of the analyte toward the UME, and a minimum detectable concentration of 0.1 fM was observed for human platelets with fixation. In contrast to such blocking methodology, the redox activity of bacteria themselves was exploited to regenerate a reduced (or oxidized) redox mediator at the Pt UME, thus providing a certain level of current amplification and with the advantage of discriminating live and dead bacteria [72]. Moreover, proof-of-principle identification of differently redox-active bacteria was provided based on the mediator oxidation/reduction rate.

Electrochemical imaging platforms based on (i) microelectrodes array chips with deposited biological specimens or (ii) scanning probe techniques have emerged as promising complementary candidates or low-cost alternatives of standard methods for tissue imaging [73]. In particular, in the latter approach, a SECM can be used to precisely position the micro- or nanometer-sized probe in close proximity of the site of interest and create maps of biomarker distributions, live cells, or other active sites. However, due to the irregular and dynamic shape of tissue samples, complex experimental setups are required to deconvolute topological artefacts from the electrochemical information. In contrast, the use of soft and flexible probes that can be gently brushed in a weak contact mode on the tissue has been proposed, demonstrating high-resolution electrochemical visualization of biomarkers in tissues by simply positioning the soft tip at a constant distance. Biodistribution of injected nano drug carriers (graphene oxide (GO) nanoribbons), melanoma biomarkers, and redox-active proteins in large and thick animal as well as human tissue was reported [74–76].

Lastly, amperometric microsensors can be incorporated into flow detection cells and utilized in combination with miniaturized chromatographic system, which benefit from smaller dead volumes and decreased consumption of both the sample and the mobile phase. Electrochemical detection in high-performance liquid chromatography (HPLC) using CF microelectrodes has been demonstrated with a model set of phenolic acids [77] and applied to 8-oxo-7,8-dihydro-2’-deoxyguanosine (8-oxodG) analysis in biofluids, reaching sub-nanomolar detection limits [78]. Furthermore, Au-modified CF microelectrodes were applied as voltammetric sensors in the liquid chromatography-pulsed amperometric detection (LC-PAD) of cysteine [79].

### Single cell metabolism monitoring

Since the very first examples in the nineteenth to twentieth centuries, the increasing interest in downsizing the probe dimensions was to a large extent driven by the scientists’ need to answer biological questions. While cellular heterogeneity in terms of structure, composition, and functionality is a fundamental principle of cell biology, its developmental and environmental origins can be investigated using single cell analysis [80]. The development of automated microfluidic–based culture systems and platforms, e.g., lab-on-a-chip or micro-total-analysis-system (µ-TAS) technologies including microseparation or electrophoresis, has significantly facilitated single cell manipulation. For instance, the integration of planar Pt microelectrodes within a µ-TAS enabled the measurement of the glucose consumption rate in single cardiac myocytes (0.211 ± 0.097 mM min^−1^ (*n* = 7) in Tyrode’s solution with 5 mM of glucose) [81]. Moreover, electrochemical detection of AA in single liver cancer cells was carried out using Pt NP–modified CF microelectrode as the electrochemical detector in a capillary electrophoresis platform [82]. On the other hand, advances in the fabrication of nanoelectrodes have led to unprecedented opportunities to carry out highly resolved and localized electrochemical experiments with minimal disturbance of the living cell functions [15]. Platinized carbon nanoelectrodes (5–200 nm radius) were used to monitor O_2_ consumption inside and outside a neuron, and minimal cell function perturbation upon repeated insertion and retraction of the nanoelectrode inside a single melanoma cell was demonstrated [83]. Overall, the determination of reactive oxygen and nitrogen species (ROS, RNS) from single cells has been attracting significant attention due to their role in the development and evolution of tumors and neurodegenerative diseases. However, selective quantification of ROS/RNS is challenging using traditional detection methods due to their fast release through sudden bursts and their ultra-low amounts. Pt/Pt black nanoelectrodes (40–60-nm radius) were reported for ROS and RNS detection at + 850 mV vs. Ag/AgCl inside macrophages [84]. Needle-type CNEs were etched to form nanocavities and functionalized with Prussian blue (PB) for amperometric detection of H_2_O_2_ at low applied potential [85]. Transient intracellular H_2_O_2_ levels were monitored upon application of − 150 mV vs. Ag/AgCl/3 M Cl^−^ during penetration-induced oxidative outbursts in murine macrophages [86]. ROS and RNS were also detected by potential-step chronoamperometry inside breast cancer cells using platinized CNEs, obtaining mean production rates of ∼35 nM/s and ∼90 nM/s for O_2_•^–^ and NO•, respectively [87].

## Micro- and nano-sized impedimetric sensors

Impedance measurements allow to probe several interfacial phenomena in a non-destructive, label-free manner upon superimposing a small sinusoidal potential perturbation at the working electrode over a wide frequency range, in the presence or absence of a redox mediator (Faradaic and non-Faradaic mode, respectively). Acquisition of the corresponding oscillating current leads to the generation of an impedance spectrum that can be interpreted using equivalent circuits to model the electrochemical interface under investigation. Effects of solution resistance, charge transfer resistance, double layer charging, and diffusional processes can be explicitly observed. In particular, due to the high sensitivity of this technique to any change occurring at the electrode surface, micro- and nano-sized probes have been developed for electrochemical immunosensing applications and live cells monitoring [88, 89].

### Immunosensors

**Fig. 2:**
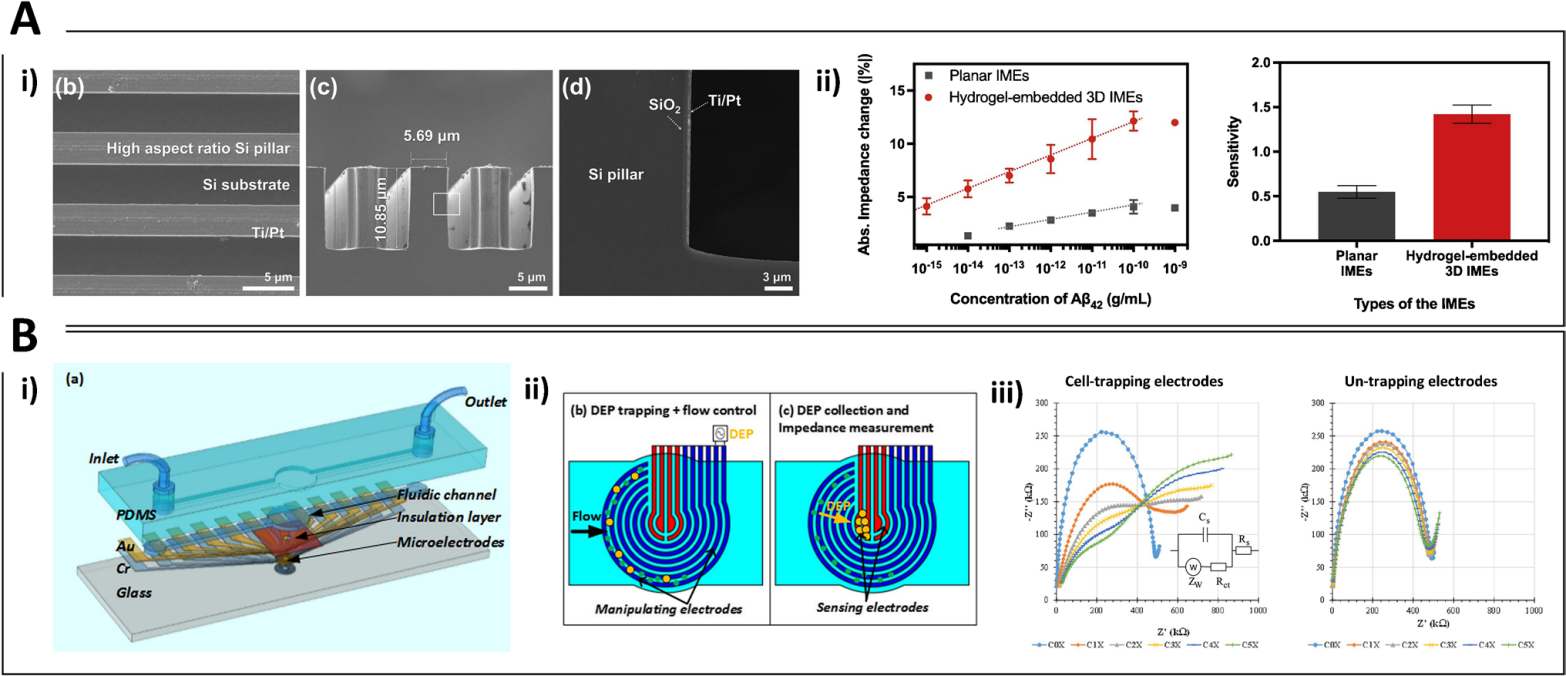
**A** Three-dimensional interdigitated microelectrode (3D IME) biosensors for proteins detection. i) From left to right: SEM images showing a top-down view of the 3D-IMEs; a cross-sectional view at the fingers of the 3D IMEs; a magnified view of the white box depicted in (c). ii) (Left) Absolute impedance change acquired from detecting various concentrations of Aβ42 with planar IMEs (gray) and hydrogel-embedded 3D IMEs (red). (Right) Sensitivities calculated by linear regression with the 95% confidence interval. Reprinted with permission from [96]. Copyright 2020 Elsevier. **B** Impedance detection integrated with dielectrophoresis (DEP) enrichment platform for lung circulating tumor cells in a microfluidic channel. i) Design of the microfluidic device for the manipulation and detection of target cells. ii) Combination of cells sample injection and DEP application to trap cells at the outermost circular electrodes Target cells that are represented by the yellow dots (like as A549). Non-target cells are represented by the green dots (like as RBC); target cells are collected in the center of the working chamber due to the DEP effect and by size difference, and trapped onto the pair of the left sensing electrodes. Then, the impedance measurement identifies the presence of the target cells. iii) EIS spectra for A549 cells concentrated into the sensing region of the microchip in sucrose buffer solution, in comparison between two pairs of central electrodes (cell-trapping and un-trapping electrodes) at different numbers of trapped cells: (C0X) 0, (C1X) 10, (C2X) 20, (C3X) 30, (C4X) 40, and (C5X) 50 cells, respectively. Reprinted with permission from [103]. Copyright 2018 Elsevier

Nucleic acid detection and particularly DNA hybridization is clinically relevant for the investigation of mutations and drug resistance genes. Direct detection of a specific gene from the antibiotic-resistant bacterium MRSA (methicillin resistant Staphylococcus aureus) was carried out using photolithographically fabricated Pt microdisk electrodes modified with a mixed film of 6-mercapto-1-hexanol and a thiolated single stranded DNA capture probe. Using a Faradaic impedimetric approach, the authors demonstrated the real time measurement of oligonucleotide binding events after nanomolar target addition caused by the increase of the charge transfer resistance (R_CT_) [90]. Faradaic electrochemical impedance spectroscopy (EIS) was applied for the direct detection of DNA hybridization using an array of Au microelectrodes modified with CVD-grown graphene. ss-DNA capture probes were immobilised on the electrodes surface simply by π-π interactions of their aromatic groups with the graphene monolayer. This interaction is lost after hybridization as the duplex leaves the electrode surface, thus leading to an overall decrease of the electrode impedance. The impedance at 0.1 Hz was proportional to the concentration of complementary DNA strands between 5 pM and 5 nM [91]. Impedance changes due to biorecognition at a microelectrode surface can be enhanced using IDEs structures leading to lower detection limits and improved S/N ratio. An electrochemical immunosensor based on Au IDEs for the detection of the organophosphorus pesticide chlorpyrifos was realised upon immobilisation of anti-chlorpyrifos monoclonal antibodies on the Au surface using protein A. A linear relation between impedance change recorded in Faradaic mode and chlorpyrifos concentration was found in the range 1–10^5^ ng/mL, with a detection limit of 0.014 ng/mL [92]. Incorporation of microelectrodes within a microfluidic platform has the potential advantages of multiplex sensing and integration of separation steps for increased S/N towards lab-on-chip technology. A recent report has compared the effect of static drop condition and microfluidic flow condition on the capacitance response of a Au NP–modified IDEs sensor detecting cancer antigen-125 (CA-125), showing slight performance losses due to shear stress caused by the microfluidic flow during the antigen–antibody binding. To overcome this drawback, the authors suggested to treat the surface of the microchannel to control its hydrophilicity and reduce the shear effect [93]. The IDEs also holds the flexibility to modify the sensing surface either using the well-assessed thiol-Au surface chemistry or through silanization of hydroxyl groups on the glass/SiO_2_ in the gaps that separate the microelectrodes [94]. The interspace between IDEs was exploited as the biorecognition site for impedimetric detection of the PSA (detection limit of 0.51 ng/ml in serum) [94] and plasma Aβ levels down to 0.1 pg/ml [95]. Amyloid beta 42 (Aβ_42_) peptide as well as PSA detection was reported using a 3D hydrogel matrix (Fig. 2A) to increase the number of antibodies immobilized in the interspace [96].

### Live cell monitoring

Thanks to the label-free, non-destructive analysis conditions, impedance-based methods have also been widely exploited using micro-/nano-sized electrodes supporting or interacting with living cells.

The impedance of carbon nanopipettes was monitored for probing single osteosarcoma cells, demonstrating significant differences in signal magnitudes between nuclear and cytoplasmic penetration. Decrease in capacitance and increase in the electrode resistance were found to relate monotonically to the cell penetration depth, thus offering a quantitative strategy for tip positioning [19]. Characterization of cell electrophysiology can be carried out based on impedance variations, which in turn depend on frequency, cell coverage, shape and growth, integrity of the cell membrane, and nature/size of the cell-electrode cleft [89]. After seeding onto a micro-electrode network at the bottom of a culture dish, cell populations that differ regarding cell density, proliferation rate, adhesion characteristics, or cell morphology can be distinguished by the impedance readout, thus having the major advantage of online monitoring if compared to standard endpoint assays. This was demonstrated studying different neuronal cell lines where adhesion, proliferation, cell death kinetics, and neuroprotective effects were detected using impedance recordings [97], with the real-time evaluation of targeted tumor therapies in HeLa cells seeded onto interdigitated electrode structures integrated into the bottom of a 96-well plate, where impedance variations were correlated with cells viability [98]. More recently, impedance monitoring of fibroblasts response to phenolic compounds under H_2_O_2_-induced oxidative stress was carried out in a 16-well E-plate with microelectrode arrays at the bottom of each well. The authors chose the area under the impedance curve to monitor changes due to cell adhesion and number following addition of 12 different antioxidant phenols, demonstrating potential for future high-throughput screening applications [99]. Lastly, impedance-based approaches for the real-time monitoring of proliferation and viability have also been integrated with microfluidic systems, allowing manipulation and study of 3D cultures of human oral cancer cells during perfusion of anti-cancer drugs [100], assessment of the impedance changes resulting from T cell activation with single-cell resolution using an array of vertical Pt microelectrodes [101], leukocytes sorting and impedance-based profiling to determine cell activation in type 2 diabetes mellitus using whole blood [102] and, in combination with dielectrophoresis, detection of lung cancer cells [103] (Fig. 2B).

## Micro- and nano-sized potentiometric sensors

Due to the abundance of literature concerning micro- and nano-scale sensors based on amperometric and impedimetric transduction reported in the last decade, the previous sections have been focused on cutting-edge functionalization strategies and especially advanced applications of these devices. The great success of these classes of sensors can be related both to historical reasons and to the massive impact that miniaturization has in improving mass transport and resolution of the measurement. Differently, micrometric and nanometric potentiometric sensors are overall scarcely represented. It is worth to note that the macro-sized counterpart is largely employed in almost any kind of academic or industrial scenario, and, in the last years, millimetric all solid-state potentiometric sensors have been attracting more and more interest in technologically challenging fields, such as paper-based and wearable devices. Obviously, the reason for such a limited number of publications about micro-/nano-potentiometric devices is not the lack of interest in miniaturizing these sensors, but it is rather related to the complex construction of reliable and stable potentiometric micro-/nano-probes. For these reasons, the first part of this section is dedicated to the main technological challenges and solutions concerning the miniaturization of potentiometric probes, while in the last paragraphs some successful applications are discussed thoroughly.

### From micropipette ISEs toward micro solid-state ISEs

Ion-selective electrodes (ISEs) are fundamental analytical tools to obtain information about the activity/concentration of ionic species. Researchers have long been recognizing the importance of studying the spatial distribution of ionic species consumed or released on the active sites of various solid/liquid interfaces. This can be the case of living cells, which exchange ions with the surrounding media, or corrosion science, where localized corrosion phenomena take place on metal surfaces. Miniaturization of the ISE allows to directly measure ions activity in real time while achieving extremely localized spatial information that is not accessible to any other analytical technique. However, the internal structure of ISEs and reference electrodes employed in potentiometric measurements typically comprises a liquid filling that guarantees the stability of the electrochemical potentials of interest. If this does not represent a problem in macroscopic conventional electrochemical devices, it has historically represented the major limitation towards miniaturization.

The first miniaturized ISEs were developed using glass capillary microelectrodes with liquid ion-selective membranes and inner filling solutions. The preparation and application of this type of electrodes are well described by Ammann and Thomas in their books [104, 105]. Glass capillary ISEs can be fabricated with many different types of glass, shapes, and geometries, constructing single-, double-, and multi-barrelled micropipettes, reaching dimensions down to 0.045 µm despite the complex preparation of microelectrodes with predetermined tip diameters. A key step in the preparation of these devices is the silanization of the glass surface, to make it lipophilic and facilitate the filling with an organic membrane solution. Capillary glass ISEs have been employed to simultaneously detect multiple parameters (membrane potential, cell membrane resistance, ion activities), depending on the number of single-barrelled microelectrodes employed or on the number of barrels that make up a multi-barrelled microelectrode. Micro ISEs for pH, Na^+^, K^+^, Ca^2+^, Mg^2+^ can be successfully prepared, showing high range of linearity and Nernstian sensitivity with acceptable selectivity toward other ions. Typical problems in the use of micropipette ISEs with an internal filling solution include the increased electrical resistance and the necessity of special electrical shielding during the measurement. Moreover, osmotic pressure originating from differences in the ionic strength of samples and the inner filling solution results in net water transport into or out of the inner filling solution, which can lead to large volume changes and delamination of the sensing membrane [106]. Other disadvantages are the limited lifetime of the devices, which is typically 1 day, and the fragility of the glass capillary that completely hinders its use in all the applications where the sample under study might move. Lastly, the detection limit and selectivity of such electrodes are limited by the flux of primary ions from the ion-selective membrane and inner filling solution that contaminates the near boundary layer of the solution that is in contact with the ion-selective membrane. Besides all these points, next-generation devices require robust, miniaturized ion-sensing systems that can be integrated with electronic control, measuring, and data acquisition units. Therefore, it is highly desirable to replace conventional micro-ISEs with all-solid-state potentiometric sensors, where a solid contact is formed between the sensing membrane and an electron-conducting substrate to replace the liquid contact.

The performance of all solid-state ISEs largely depends on the solid contact that mainly employs conducting polymers and/or high-surface-area nanostructured materials [107, 108]. The conducting polymer addresses the issue of charge carrier mismatch in the ion-selective membrane (ions) and the electrode (electrons). Moreover, carbon materials such as graphene, graphene oxide, and carbon nanotubes are often used to increase the double-layer capacitance at the membrane–electrode interface to stabilize the electrode potential in coated-wire ISEs [109]. Advances toward improved solid-state ion-selective electrodes have been well described in several excellent reviews [109–112]. The first applications of solid-contact ion-selective microelectrodes (ISMEs) regarded SECM, where these devices were employed as tips for the detection of a variety of ions. K^+^ selective microelectrode using Pt, Au, or CF by casting the ion-selective membrane were developed, based on polyvinyl chloride (PVC) deposited on an electrochemically polymerized film of polypyrrole [113]. Bakker and co-workers described a Ag^+^ selective solid-contact microelectrode of 100–200 µm diameter with nanomolar detection limit. A drawback of their system was the size of the measuring point (more than 300 µm), which cannot provide sufficient spatial resolution for localized measurements [114]. An interesting approach to reduce the size of solid contact ISE was proposed by Jun Ho Shim and co-workers [115], who used a cone-shaped glass nanopore structure to construct a layered configuration for chloride or pH ISEs. The authors employed a Pt disk of 500 nm radius and deposited a Ag/AgCl layer within the pore. The Ag/AgCl layer-coated ISE was used as a highly selective Cl^−^ probe in SECM experiments to map the ion flux through a micropore. To demonstrate the versatility of their approach, the authors also prepared an ISE based on an IrO_2_ layer at the base of a glass nanopore electrode that exhibited a highly sensitive response to variations in pH (79.7 ± 2.3 mV/pH) and could be used for 3 weeks. In another contribution, a novel type of solid-contact ionophore-based ion-selective microelectrodes for Mg^2+^ and pH was realized showing stable potential and fast response that are essential properties for the practical application of microelectrodes for localized scanning measurements. The microelectrode was based on an insulated needle-shaped metallic wire with an exposed apex. The ion-to-electron transducer was made of poly(3-octylthiophene-2,5-diyl) (POT) and placed between the ion-selective membrane and the metallic tip [116].

### Miniaturization and integration of the reference electrode

In all the applications discussed in the previous paragraph, the ISE was of miniaturized size but a traditional macroscopic RE was employed, and we think that this is a point that deserves attention when speaking about the miniaturization of ion-selective electrodes. The RE is an essential component of any potentiometric device and its performance profoundly influences the response of the whole electrochemical system. The required stability and precision in fact largely depend on the mode of operation. In amperometric measurements, the potential of the working electrode must be accurately controlled; however, a small shift in the RE potential is not a big deal and in fact pseudo-reference electrodes (non-polarizable electrodes such as Ag/AgCl, and even polarizable electrodes such as Pt, Au and carbon based) are often used under limiting conditions. On the other hand, in potentiometry, the shift in the RE potential directly affects the reading of the potential of the indicator electrode and causes a serious error. Therefore, the approach of using a pseudoreference electrode is incompatible with ISEs and, currently, commercially available reference electrodes (in the cm size) are used together with many miniaturized potentiometric devices.

If the target application requires the use of a miniaturized RE and not only of a miniaturized probe, several strategies can be followed. A first approach consists in the miniaturization of the conventional liquid-junction Ag/AgCl electrode with an internal filling solution. In a Ag/AgCl reference electrode, the potential depends, as described by Nernst equation, on the Cl^−^ concentration that is maintained constant by a concentrated KCl filling solution, which also helps to limit the magnitude of the liquid junction potential. As the volume of the filling solution decreases, it is more challenging to meet these requirements and the incorporation of a RE in a micrometric device results problematic. A RE capable of working in a completely dry state would be desirable, and many groups have attempted to develop so-called solid-state reference electrodes [116–118]. In these systems, the liquid filling solution of conventional REs is replaced by a wide variety of solidified reference electrolytes (melted, gelled, embedded in a polymer matrix, etc.) [119, 120]; however, some drawbacks remain such as the often-limited lifetime and the need to recondition the electrode frequently. Another approach to solve these issues employs polymeric membranes doped with ionic liquids (ILs), in which a sample-independent interfacial potential is defined by the limited degree of partitioning of IL ions into the sample and therefore there is no need for reconditioning [121–124].

### Monitoring of corrosion processes

An important application of micrometric ISEs is to image the concentration of ionic species during corrosion processes. In these applications, the ISE is typically used as SECM tip in combination with a conventional macro-sized reference electrode, to provide a reliable potential measurement. In this regard, most applications concern the measurement of Mg^2+^ concentration, as first introduced by Souto et al. [125]. The performance of a new solid-state Mg^2+^ ISME was compared with that achievable with a conventional liquid contact ISE having the same tip dimension (200 µm), using them as probes to perform SECM experiments on a model corroding system [126]. The internal contact of the solid-contact ion-selective microelectrode was a 33 μm diameter CF that was coated by electrodeposition with poly(3,4-ethylenedioxythiophene) (PEDOT) prior to its insertion in a glass microcapillary. The experiments showed that the solid-contact ISE exhibited smaller internal resistance, greater stability, and a faster response time compared to one with a conventional liquid contact. These features allowed to collect SECM images with a high spatial resolution needed to study early stages of localized corrosion. This work highlighted the role of the electrode resistance in the ISME performance, where the smaller the resistance, the lower the noise associated to the measurements, thus allowing to perform a more reliable detection. Another example of Mg^2+^ solid-state microelectrode was published by Salleh et al., who fabricated Mg^2+^ ISMEs using CFs of 7 µm diameter as the electrode support [127]. Once coated by PEDOT, the CF was inserted into a borosilicate glass capillary (outer diameter of 1.5 mm and inner diameter of 20 μm) filled with the Mg^2+^ ionophore cocktail. The Mg^2+^ ISME was used as SECM probe for measuring the local Mg^2+^ concentration above a dissolving Mg specimen during both free and galvanic corrosion. The size of the ion-selective microelectrode was further reduced by P. Dauphin-Ducharme et al., who realized a 500 nm potentiometric micro-Mg^2+^ sensor possessing a large dynamic range and a good selectivity towards Mg^2+^ [128]. This sensor, that could be prepositioned with high resolution above the substrate, was used to monitor Mg^2+^ release from three types of corroding surfaces and showed a high sensitivity to small microstructural variations, thus allowing the real-time tracking of Mg^2+^ release through the stages of the corrosion process.

### Monitoring of ion fluxes in cells or other matrices

**Fig. 3:**
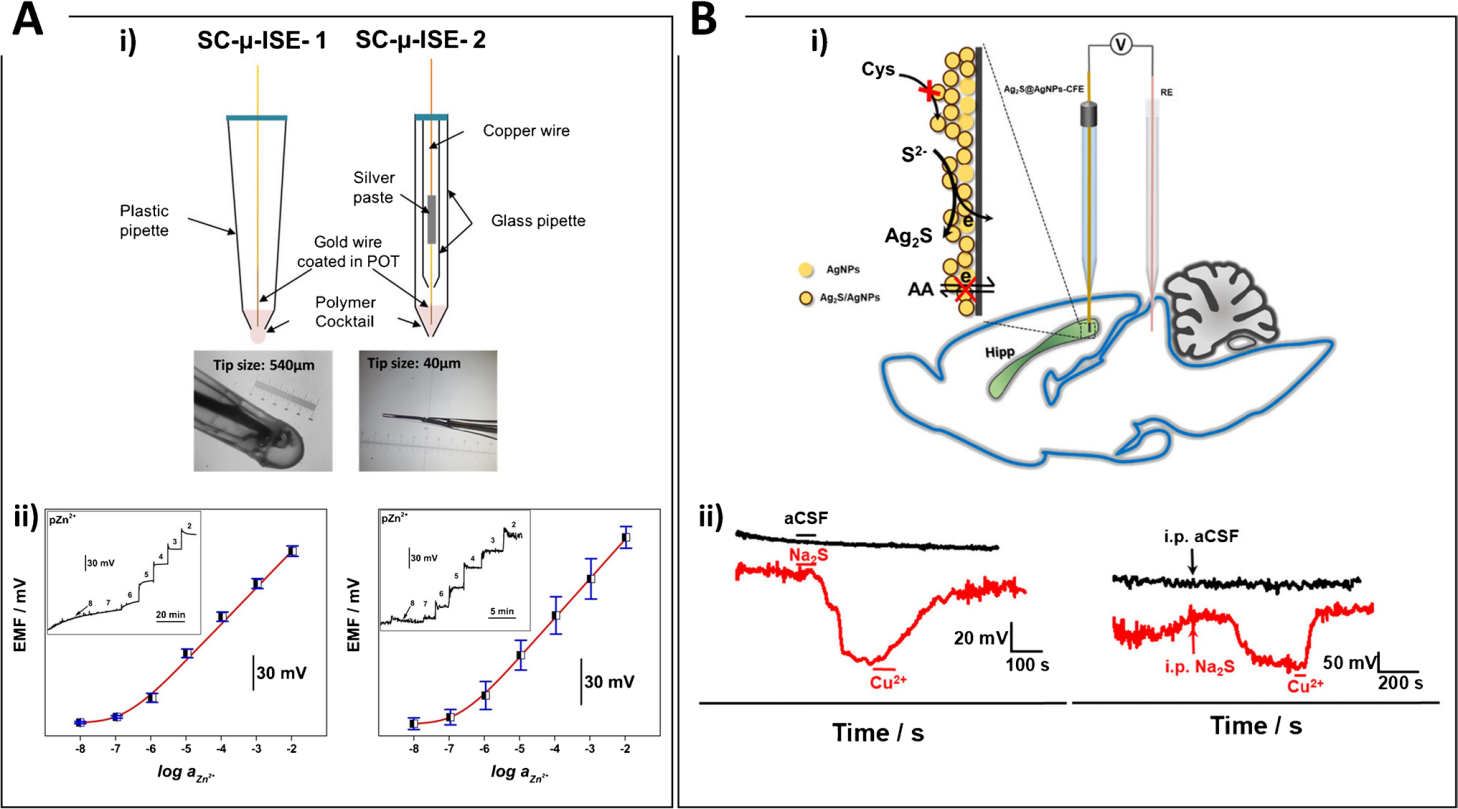
**A** Needle-type ion-selective microsensors for in situ determination of foliar uptake of Zn^2+^ in citrus plants. i) Diagram and photograph of SC-μ-ISE 1 (left) and SC-μ-ISE 2 (right). ii) Zn^2+^ calibration curves obtained with (left) SC-μ-ISE 1 and (right) SC-μ-ISE 2. (Inset: recorded potential time traces of respective SC-μ-ISEs). Reprinted with permission from [129]. Copyright 2017 WILEY–VCH. **B** Ag_2_S/Ag nanoparticle microelectrodes for in vivo potentiometric measurement of hydrogen sulfide dynamics in the rat brain. i) Scheme of the H_2_S measurements in rat brain with Ag_2_S/Ag NPs/CFE. ii) In-vivo OCP response obtained in the hippocampus of rats: (left) during local microinjection of pure artificial cerebrospinal fluid (aCSF) (black curve), aCSF containing 100 μM Na_2_S, and aCSF containing 400 μM Cu^2+^ labelled in the figure (red curve); (right) during intraperitoneal injection of pure aCSF labelled with a black arrow (black curve), aCSF containing 40.8 mg/kg Na_2_S, and aCSF containing Cu^2+^ 400 μM labelled in the figure (red curve). Reprinted with permission from [134]. Copyright 2021 American Chemical Society

An important aspect concerning the application of an ISME to the study of ion fluxes in cells or other micrometer-sized samples is the evaluation of how the tip dimension will affect the measurement and up to what size the device can be scaled down. Church et al. tried to answer these questions, developing Zn^2+^ solid-contact micro-ISEs with different tip dimensions to study ion-transport processes in the foliage and roots of citrus plants [129] (Fig. 3A). The first electrode was prepared by employing a commercially available micropipette tip (0.540 mm diameter), where a Au wire (0.20 mm diameter) coated with POT was introduced and sealed from the top with a hot melt adhesive. The zinc ionophore cocktail was inserted through capillary action and a round-like membrane formed at the end of the tip. The preparation of the electrode having the smaller tip dimension was more laborious: borosilicate glass micropipettes were pulled horizontally using a micropipette puller, to create a tip diameter between 30–100 µm. An important step, when trying to lower the electrode dimensions, is to guarantee a good adhesion of the ion exchange membrane to the glass of the electrode, to prevent the aqueous electrolyte solution from finding a pathway along the glass and short-circuiting the sensor, and, therefore, the glass inner surface was silanized. The tip was then dipped in the Zn^2+^ ion-selective membrane cocktail for 10 s, and a 100 µm POT-coated gold microelectrode was positioned within the membrane. An important finding was that the tip size, ranging between 40 and 540 µm, did not modify the sensor’s sensitivity, but only slightly increased the noise associated to the measurement, thus affecting the measurement accuracy. It is important to highlight that all the measurements were conducted in a faraday cage using a high input impedance acquisition system. The electrode with the smallest tip dimension showed a detection limit (LOD) of (3.96 ± 2.09) × 10^–7^ M, whereas the larger one of (2.83 ± 0.47) × 10^–7^ M. For this experiment, a standard macro-sized RE was used. A very recent example where both the indicator and the reference electrode were miniaturized reports the fabrication of a pH nanosensor for single intracellular measurements, consisting of two solid-contact carbon nanopipette electrodes whose tip dimension was 800 nm diameter, tailored to produce both the indicator (pH nanosensor) and RE [130]. The pH nanosensor was composed of a two-layer structure: a carbon film, to provide large conductivity to the electrode substrate and a solid contact to ensure a proper ion-to-electron transduction, and the proton-selective membrane. The ISE displayed Nernstian sensitivity in the pH range of interest (6–8.5), a fast response time (< 5 s), and a low medium-term drift (0.7 mV h^−1^). The use of a miniaturized RE allowed the authors to investigate how the position and configuration of the RE affects the measurements, studying three different configurations in which (i) both the indicator and reference electrodes were located inside the same cell, (ii) each of them inside two neighbouring cells, or (iii) the indicator electrode inside the cell and the reference electrode outside of (but nearby) the studied cell. The experiments revealed that the position of the RE did not influence the measurement. Another work employing a micro-solid-state RE was published by Gallardo-Gonzalez et al., who developed an all-solid-state and highly selective amphetamine microsensor consisting of four ISEs of 0.64 mm^2^ surface and two solid-state REs of 0.13 mm^2^ surface [131]. The device showed a selective and Nernstian response with a slope of 60.1 mV/decade within the concentration range 10^−5^ M to 10^−3^ M amphetamine, with a LOD of 12 µM and a response time shorter than 10 s.

### In vivo applications

An important field of interest for ISME application is in vivo monitoring of ion fluxes. In this case, both the indicator and the reference electrode require micrometric size. A solid-state ISE was developed for real-time monitoring of the extracellular Ca^2+^ in the brain of living rats [132]. The authors employed hollow carbon nanospheres (HCNs) as transducing layer and solid contact, demonstrating that HCNs can improve the signal stability of the solid-state ISEs as a result of the unique hollow structure and of their surface hydrophobicity. The ISEs employed a single CF of 7 μm diameter as the conductive material that was inserted in a glass capillary, whose tip was 30 − 50 μm diameter. The authors performed in vitro and in vivo measurements employing also a tissue-implantable Ag/AgCl microelectrode, demonstrating a high measurement stability and selectivity against the species endogenously present in the brain, as well as tolerance against O_2_ and light. Another Ca^2+^ ISMEs for in vivo applications was fabricated employing acupuncture needles having a tip diameter lower than 80 µm [133]. The ISE was realized by coating the acupuncture needle tip, which was previously modified with PEDOT:polystyrene sulfonate (PSS) as solid contact, with a calcium ion-selective membrane. The device showed a Nernstian response toward Ca^2+^ in the range from 1.0 × 10^–6^ to 3.1 × 10^–3^ M and a detection limit of 1.2 × 10^–7^ M. It was used for in vivo monitoring of the calcium changes in rat cerebrospinal fluid, demonstrating the compatibility of such device for in vivo monitoring with high temporal resolution and flexibility. A Ag/AgCl reference microelectrode was used and kept at 5 mm distance from the indicator electrode. Recently, a very interesting example of in vivo application was reported by Zhang et al. (Fig. 3B), who monitored the in vivo dynamics of H_2_S in the rat brain using a solid-contact ion-selective microelectrode based on a CF microelectrode coated by Ag_2_S/Ag nanoparticles [134]. The device exhibited a selective response toward H_2_S with Nernstian sensitivity in the range of 2.5–160 μM, with a detection limit of 0.8 μM, and was able to detect H_2_S and pH variations in the rat brain during local microinfusion of Na_2_S.

## Micro- and nano-sized sensors based on a transistor architecture

Transistor-based sensors were introduced by Bergveld in the early 1970s and were derived from metal–oxide–semiconductor field effect transistors (MOSFETs) [135, 136]. It was found that ions can be detected by a MOSFET as long as its architecture is changed by removing the metal gate and inserting the gate oxide in an aqueous solution along with a reference electrode. For this reason, such devices were called ion-selective field effect transistors (ISFETs). Starting from Bergveld’s experiments, the research and commercialization of transistor-based chemical sensors were focused on those application fields wherein the conventional electrochemical sensors, such as the glass electrode, are not convenient or cannot be used. In particular, they have been successfully employed in all those determinations requiring low sample volumes and extremely high degree of miniaturization.

Transistors are electrical devices where the electrical current flowing across a semiconductor, whose length is defined by the source and drain terminals, can be modulated upon application of voltage or current through another pair of terminals (usually gate and source). In an electrical circuit, transistors are used to amplify or switch electronic signals and electrical power. If one or more chemical species are able to interact with the semiconductor or other elements of the transistor architecture, their presence will affect the transistor operation (current–voltage relationships or source-drain current) and thus will be detected. The difference to a chemiresistor stems from the presence of a gate electrode by which one can apply fixed voltages or voltage sweeps. The analyte detection can occur via several possible mechanisms, such as by creating traps, acting as a dopant, imposing resistive interfacial barriers, changing the existing intermolecular interactions between molecular subunits in semiconductor and dielectrics, or inducing an electric field that perturbs the effective gate voltage. These interactions can be exploited for the design of sensors that are able to detect analytes both in liquid and gas phases. Moreover, the signal can be directly amplified by the acquisition unit, thus avoiding the amplification of electrical noise due to additional inductances. The semiconductor material can be both inorganic, such as doped Si or Ge, and organic. While the production of inorganic materials is a mature technology with many examples of commercialized devices, transistor sensors based on organic semiconductor materials are nowadays at a research stage but hold great potential towards the production of flexible sensors for physiological monitoring and wearable applications. This section focuses on ISFET, graphene field effect transistor (GFET), and organic electrochemical transistor (OECT)–based electrochemical micro- and nanosensors, highlighting major advantages and disadvantages related to each transistor architecture in comparison with the previously described conventional electrochemical setups, and encompasses the most recent applications in the field of electrochemical sensing.

### ISFET sensors

**Scheme 1:**
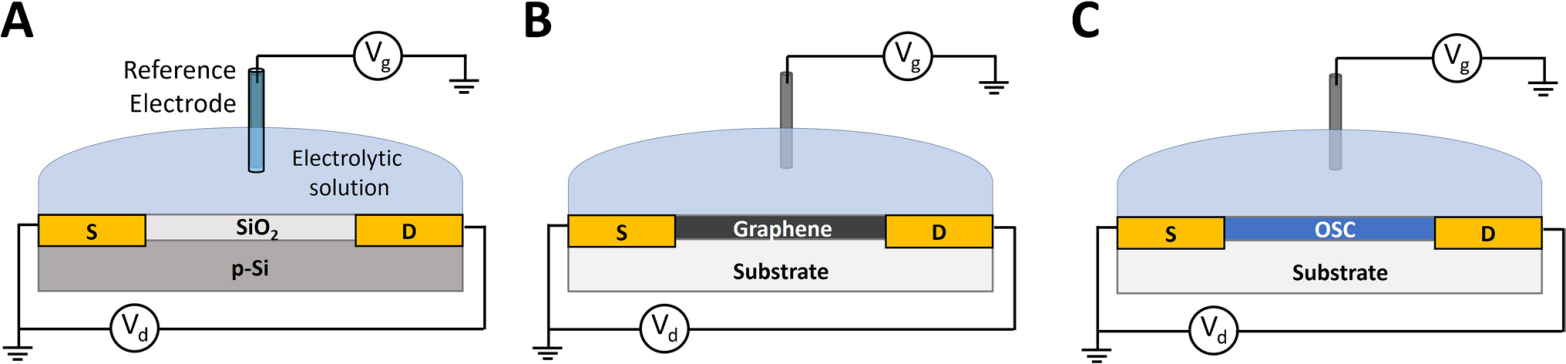
Basic device structure of **A** ISFET,** B** GFET, and** C** OECT (OSC: organic semiconductor)

ISFETs represent a class of FETs capable to detect chemical species in different media. As already mentioned, the ISFET configuration (Scheme 1A) for sensing was derived from the MOSFET architecture. MOSFETs include four terminals (source, drain, body, and gate). Source and drain are connected to Si n^+^–doped regions, which are separated by the body region that is Si p^+^–doped and is connected to the base. The gate is a metal plate that is insulated from the body of the transistor by a thin layer of SiO_2_. The application of a potential to the gate electrode (with respect to the source, which is connected to the ground) controls the flow of e^−^ from source to drain by affecting the size and shape of a “conductive channel” created between the two terminals. While positive gate voltages lead to an enhancement of the drain current (I_d_) because the channel is enlarged, negative gate voltages deplete e^−^ and decrease the channel size. By far, the most widely studied chemically sensitive FET is the ISFET, which can be seen as a second-generation ISE. In the latter, the measured potential difference between reference and indicator electrodes is typically amplified using a pH-meter circuitry. Integration of the ion-sensitive membrane and the solid-state amplifier and mitigation of the contact problems between the two electrodes have been accomplished by reducing the size of the whole assembly and making the internal conductor shorter and shorter, until the sensing material is directly deposited on the gate electrode [137]. This device is called ISFET. Its architecture differs from a MOSFET because the metal gate electrode is replaced by a reference electrode inserted in an aqueous solution that is in physical contact with a thin sensitive layer deposited on the transistor channel. For this reason, the ISFET operational mechanism can be discussed starting from the theoretical description of a MOSFET [137]. Overall, ISFETs work thanks to a variation of threshold voltage due to the variation of surface potential at gate/sample interface, in analogy with potentiometric sensors. ISFETs usually exhibit a sub-Nernstian sensitivity and Bergveld’s group has thoroughly studied this effect in pH measurements, demonstrating that the Nernstian sensitivity represents the limit that an ISFET can reach in the best conditions, because different phenomena affect the gate action on I_d_ [135]. However, the transistor structure can be designed to overcome this limit through signal amplification occurring during the measurement. For example, dual gate transistors have an architecture with an additional gate that is placed on the opposite side of the channel with respect to the sensitive layer in contact with the sample. This structure enhances the sensitivity due to capacitive coupling involving the sensing layer and the thick bottom gate. It is worthy to note that some studies demonstrate that, despite the enhanced threshold shift (measured from the bottom gate), the potential measured at the sensitive layer exhibits sub-Nernstian slopes [138]. Moreover, this architecture can reduce the signal drift due to charges trapping. Alternatively, in the external gate field effect transistor (EGFET) the gate region is preserved as in a standard MOSFET, the sensing area is in direct electrical contact with the MOSFET gate and the gate potential is applied through the electrolyte by the use of a reference electrode. This configuration keeps the MOSFET gate area and the chemical compounds in the sample separated, thus avoiding the signal drift that occurs in ISFETs [139]. Moreover, it allows to vary the ratio between the sensing area and the channel area. Differently from potentiometric and amperometric sensors, the transistor architecture can be used to enhance the signal transduction and pre-amplify the signal before the read-out electronics, with significant advantages in design and production of micro- and nanodevices.

The fabrication of micro-sized ISFET sensors takes advantages of CMOS technology, which is a consolidated process widely employed for the production of a large plethora of microchips and circuit since its invention in 1959. In the last decade, the research on ISFETs has increased the knowledge on pH sensors by carrying out experimental and theoretical studies involving different architectures. The high maturity of this technology is demonstrated by both the commercialization of pH sensors and their large use as sensing element in devices for indirect detection of other compounds. Different ISFET pH sensors have been commercialized to answer the market demands that cannot be satisfied by the glass electrode, such as the production of miniaturized devices for the investigation of samples with a low volume. MSFET-3330–2 pH sensor and ISFET micro pH probe (SENTRON MicroFET) are examples of commercial ISFET pH sensors with a micrometer-sized sensing area and a wide operation range (pH 2–12). Nevertheless, the most robust devices are endowed with a reference electrode with the aforementioned limitations. Moreover, ISFET pH sensors have been exploited for the design of microbiosensors for the detection of chlorpyrifos [140], influenza A virus [141], *Cordyceps sinensis* DNA [142], paraoxon, parathion and methyl parathion [143], and troponin I [144]. The interaction between the bio-element and the target compounds releases H^+^ that locally decreases the pH on the sensing area, thus making the detection possible. It is worthy to note that the reliability of the transduction principle was demonstrated by the different kinds of employed reactions. The ISFET sensor is in fact able to detect the variation of H^+^ activity stemming from enzymatic reactions [140, 141, 143] and even from DNA hybridization [142].

Moreover, the developed ISFET sensor for chlorpyrifos detection was compared with a voltammetric sensor obtained with the same chemical functionalization, showing that the ISFET configuration improves the LOD of one order of magnitude, reaching a value of 10^−10^ M [140]. The surface charge at the basis of the transduction can be generated on the micro-sized gate area when it is chemically modified to interact with different target compounds. Ionophore membranes and antibodies have been exploited for the detection of NH_4_^+^, NO_3_^−^ [145], and influenza virus [146], respectively. Jang et al. [147] proposed an ISFET sensor with a 10 µm × 10 µm sensing area for the detection of human IL-5, with an approach that is similar to enzyme-linked immunosorbent assays (ELISAs). The sandwich immunoassay exhibited a LOD of 1 pg/mL that is significantly lower than the value of conventional ELISA-based methods (1 ng/mg). Due to the fast, simple, and non-invasive measurement capabilities as well as ease of automation, micro-sized ISFETs are among the most promising tools for monitoring the metabolism or ion efflux at single or few cell level. Moreover, ISFET fabrication is fully compatible with traditional photolithographic processes, thus making them suitable for high volume production and array-based measurements for simultaneous multi-well and multifunctional assays. Walsh et al. [148] measured K^+^ efflux from cultured mammalian cells by exploiting an ISFET, wherein the gate area was coated by a polymeric membrane containing the K^+^ ionophore valinomycin. The sensor successfully detected the ionic fluxes in two cell lines characterized by different K^+^ conducting channels. Li et al. [149] proposed a graphene-based ISFET for K^+^ ion efflux sensing from living neuronal glioma cells. The results were in good agreement with the data acquired with a commercial micrometric ISFET. Sakata’s group has systematically been investigating the use of commercial micro-sized ISFETs for the study of the extracellular matrix in the nanogap between cells and a pH-sensitive gate for assessing the metabolic activities of chondrocytes [150] and the allergic responses at a mast cell [151]. A correlation between the electrical signal of the ISFET sensor and the biological activity was found, and the results were validated by in-situ monitoring of cellular metabolism with a laser scanning confocal fluorescence microscope. Imaizumi et al. [152] exploited the same commercial ISFET as Sakata’s group to study apoptosis of model HepG2 cells by continuously monitoring the pH variation generated by membrane breakdown. With the ISFET approach, the authors were able to distinguish the cause of cell death by membrane leaking from that by other organelles damage upon chemical stimuli.

**Fig. 4:**
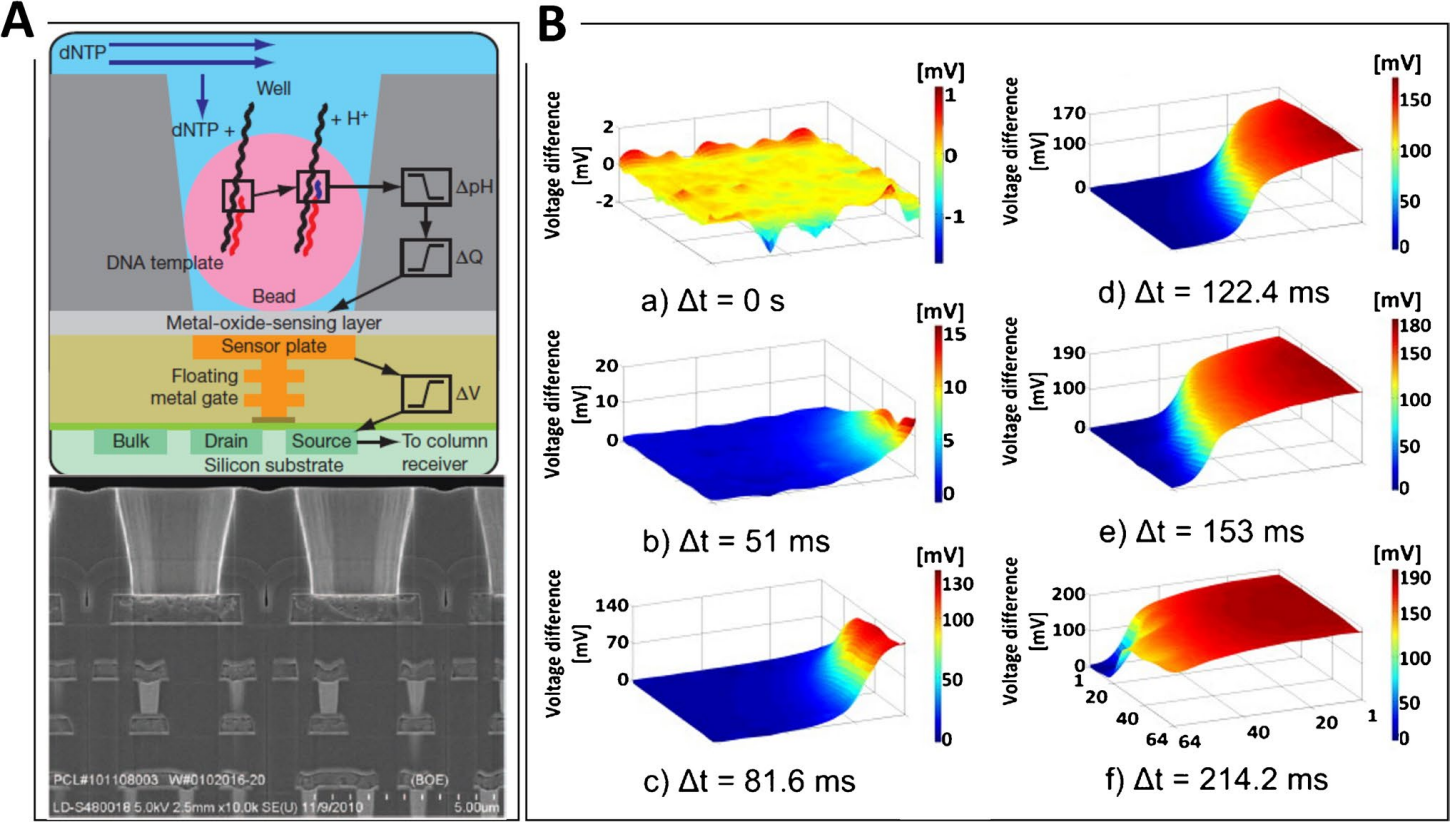
**A** Ion semiconductor sequencing of DNA: Simplified drawing of one sensing elements for DNA sequencing composed by a well, a bead containing DNA template, and the underlying ISFET sensor endowed with read-out electronics (top). The nucleotide incorporation on the growing DNA strands releases protons that vary the pH within the well. The pH change is detected by ISFET sensors. Electron micrograph showing the alignment of the wells over the ISFET metal sensor plate and the underlying electronic layers (bottom). Reprinted with permission from [154]. Copyright 2011 Springer Nature. **B** ISFET arrays: citric acid diffusion monitored by a 64 × 64-pixel ISFET array. The images exhibit the effect of injecting 1 ml 0.2 M citric acid into 1 ml 0.1 M sodium hydroxide. (a) Before the acid injection and (b–f) the progress of the acid across the sensor surface. Blue shows basic area, red shows acidic areas. Reprinted with permission from [161]. Copyright 2012 Elsevier

The CMOS technology allows for the reliable production of chips with a large number of fast, uniform, working sensors integrated with supporting read-out electronics needed for the measurements. Following the first pioneering work of Cumming’s group [153], different ISFET arrays have been proposed. Chips with millions of micrometric pH sensors are at the basis of ion proton semiconductor commercial sequencers (ion torrent and DNA electronics—Genalysis) that directly detect the H^+^ ions generated by template-directed DNA polymerase synthesis. The approach is cost-effective and competitive with respect to the optical DNA sequencers. After fragmentation and amplification, a bead containing sequencing DNA is loaded on each sensor [154] (Fig. 4A). Ion sequencing is performed massively in parallel, by providing the nucleotides on every pixel in a stepwise fashion during an automated run. When the complementary nucleotide is delivered on the sequencing primer, one or more bases are incorporated into the DNA nascent strand by the bound polymerase, leading to the hydrolysis of the incoming nucleotide triphosphate, with the release of a single proton for each nucleotide. The pH decrease is proportional to the number of incorporated nucleotides and is detected by the sensor on the bottom of each well, converted into a voltage and digitized by off-chip electronics. Rothberg et al. [154] succeeded in sequencing more than 96.8% of bacterial genomes in an individual run using a small ion chip. The per-base accuracy was observed to be 99.569% ± 0.001% within the first 50 bases and 98.897 ± 0.001% within the first 100 bases. The same authors sequenced the genome of Moore, who gave his name to the well-known Moore’s law, by using a thousand individual ion chips and about one billion sensors, thus highlighting the crucial role played by sensors miniaturization. Toumazou et al. [155] proposed an improvement of this sequencing technology by using a simpler ISFET based on Si_3_N_4_ and the chemical amplification of the signal by PCR and isothermal amplification. A CMOS lab-on-chip platform for DNA sequencing has been employed for rapid, simple, and specific diagnosis of *P. falciparum* malaria as well as the identification of mutations related to drugs administration [156]. Moreover, the development of DNA sequencers has pushed the study of the ISFET applied to DNA technology to improve the transduction by the use of dual gate configurations [157], a reference field effect transistor [158] or to detect DNA methylation [159].

ISFET sensory arrays have also been produced with the purpose of tracking ionic diffusion for the potential monitoring of ion fluxes during biomineralization, intracellular transport processes, molecular self-assembly, and synthetic biology. The first time-resolved ion mapping for non-equilibrium systems based on ISFET has been reported by Nemeth et al. [160], who took advantage of an array composed by 64 × 64 pH sensors having 10.2 µm × 10.2 µm size each to follow the growth of micrometer-scale inorganic tubes and membranes. The results have been confirmed by coupling the devices with optical microscopy. A similar device has been exploited to visualize mixing profiles due to the injection of citric acid into alkali [161] (Fig. 4B). Georgiou’s group devoted great research effort to improve the time resolution of ISFET sensory arrays, also by reducing the signal drift in each pixel using a reset switch to fix a known gate potential in order to compensate trapped charges [162–165]. This platform has been used to acquire multi-ion imaging of pH and K^+^, Na^+^, and Ca^2+^ concentrations after an offline training [166]. The cost-effectiveness of scaling up CMOS technologies could play a key role in the development of non-optical ELISA tests based on ISFET sensor arrays that detect the protons from a reaction pathway designed to amplify the signal by exploiting glucose oxidase and Fenton chemistry [167]. Although the platform has not a sensing chamber for each sensor, it is able to detect C-reactive protein and immunoglobulin E down to concentrations of 12.5 and 125 pg/mL, respectively, highlighting the potentiality of this approach. Moreover, ISFET pH sensors arrays can be combined with co-located impedance and optical sensor arrays to produce a multimodal CMOS device with boosted sensing ability [168]. Finally, micro-sensors based on ISFETs are very attractive devices for wearable applications due to the possibility for ultra-miniaturization, low power operation, and reliability. Recently, a micro-sized ISFET sensor was embedded in a sweatband to produce a prototype wearable sensor for monitoring Na^+^ in sweat [169]. The experimental campaign (25 subjects) demonstrated reliable sensor operation in relevant environment, showing a correlation between [Na^+^] and subject’s internal temperature during exercise. CMOS technology has been exploited for the fabrication of a wearable chip for the detection of pH, Na^+^, K^+^, and Ca^2+^ with a sensitivity which is close to the Nernstian limit [170]. Due to an adsorbed power of 2 pW/sensor, the chip containing the sensors and the read-out electronics were powered by a radio-frequency signal. The ISFET was deposited on polyethylene naphthalate to produce a transparent and flexible pH sensor to meet the main demands linked to wearable applications, such as conformability [171]. The transduction occurs at an extended gate consisting of a SnO_2_ sensing membrane and an amorphous indium–gallium–zinc oxide semiconductor.

Although ISFET technology exhibits a high level of readiness and reliability, the stiffness of inorganic semiconductors, the use of a hardly-miniaturizable reference electrode, and harsh fabrication conditions hinder their widespread use in some advanced applications. A measurement system that operates in vivo must not damage the surrounding tissues and should therefore have mechanical flexibility and conformability matching the adjacent biological environment. At the same time, smart clothes and dressings can be endowed with sensors that must be fabricated by soft techniques to preserve textiles and other breathable materials. Organic field effect transistors (OFETs), electrolyte-gated field effect transistors (EGOFETs), and organic charge modulated field effect transistors (OCMFETs) are some FET examples that were introduced to overcome these issues by combining a flexible organic or graphene-based semiconductor and alternative configurations that do not use the reference electrode. Moreover, differently from the above-mentioned architectures that have been historically dominated by the chip-like configuration, in the last years, spearhead FET structures were investigated, wherein both the transistor channel and gate are fabricated on the tip of micro- and nanoelectrodes. PPy [172], PEDOT:PSS [173], and graphene [174] have been studied as semiconductor materials. The main advantage of this structure is the possibility to accurately position the transistor-based sensors to map concentration gradients in space around a single living cell.

While the literature describes many examples of micrometric GFETs, very few sub-millimetric OFETs have been described due to the limited development of suitable fabrication techniques and the low mobility of charge carriers in organic semiconductors, which requires the use of large-area interdigitated electrodes as the drain and source terminals of the channel to reach high current values. Kergoat et al. [175] have proposed a DNA sensor based on a water-gated OFET with a 3 × 500 µm^2^ channel made of poly [3-(5-carboxypentyl)thiophene-2,5-diyl] modified with oligodeoxynucleotides. After hybridization, the OFET could detect the negative charge associated with the target DNA strand, which affected the transistor operation. A DA potentiometric sensor has been fabricated by exploiting a 11,200 × 20 µm^2^ poly(3-hexylthiophene-2,5-diyl) (P3HT) channel combined with a millimetric Au gate as the sensing element [176]. The Au surface was modified with cysteamine and 4-formylphenyl boronic that can selectively bind DA. An OCMFET was reported as pH sensor upon Parylene C activation on the gate electrode by O_2_ plasma, thus decorating the surface with carboxylic groups that can transduce the pH signal [177]. Different gate areas were tested, and sub-millimetric devices have been used. A potentiometric sensor has been realized by taking advantage of an OFET structure composed of a 7 × 3000 μm^2^ PH3T and a Ag/AgCl reference electrode [178]. A Na^+^ selective membrane was placed between the two transistor elements soaked in two different microfluidic chambers containing the sample and the filler solution. A nanometric field-effect-transistor was exploited to produce a sensor to measure extracellular pH and adenosine triphosphate (ATP) gradients close to living cells [172]. The bare channel, made of electrodeposited PPy, showed a high sensitivity to pH and could detect ATP after modification with hexokinase. In addition to all these examples, an OECT configuration has been reported in literature, where the modulation of the current in the transistor channel, usually made of a conducting polymer, occurs due to electrochemical processes. The high transconductance values combined with a relatively high conductivity of these polymers have made the development of this technology competitive even at the micrometric level. The following discussion will be focused on GFETs and OECTs.

In electrolyte-gated transistors, the absence of the dielectric film causes higher capacitances to rise at the main transistor interfaces. Consequently, the major advantage is the low voltage operation that, together with the improved flexibility in device architecture, has been crucial for their success in bioelectronic applications. In particular, large capacitances are obtained at the electrolyte/channel interface, thanks to the sub-nanometer thickness of the dielectric due to the accumulation of ions in the interfacial region in EGOFETs (including GFETs) and the penetration of ions into the entire channel volume in OECTs. As both phenomena occur, thanks to the ions transport across the electrolyte, the transistor architecture is no longer limited by the need of having top/bottom gate electrodes to modulate the channel conductivity. Therefore, the versatility of the transistor is greatly improved in terms of fabrication methods and substrates. The electrolyte can serve as the medium to close the ionic circuit and as the sample itself directly embedded within the transistor structure, as in the case of biological fluids [173, 174].

### GFET sensors

A graphene field effect transistor (Scheme 1B) is composed of a graphene channel, deposited between the source and drain terminals, and a gate that is usually a reference or a coplanar electrode operating in an electrolyte solution. Differently from ISFETs, no insulating layer is placed between the electrolyte and the semiconductor because many interferences are avoided by the non-polar nature of graphene, which weakly interacts with the polar compounds dissolved in water [179]. Graphene is a single layer of carbon atoms arranged in a two-dimensional hexagonal lattice with high conductivity, stability and uniformity [180]. The main sensing mechanism benefits from a configuration wherein the channel is modified with a bio-recognition element that is able to bind the molecule of interest. The binding event is detected through a change in the graphene conductivity generated by the change of the local electrical field that modulates the concentration of charge carriers. An alternative detection principle exploits a variation of scattering rates in the semiconductor. Since the channel is only one atom thick, the entire depth of the channel is exposed to and affected by the modification of the electrical field occurring on the surface. Therefore, the sensing performances are boosted with respect to that of an the ISFET, wherein the bulk of the semiconductor is practically unaffected by the sensing events occurring on its surface and cannot be produced as low-defect monoatomic layer. Due to the gap-less semiconducting behavior of graphene, the I–V curves (transfer characteristics) of GFETs typically show the peculiar V shape that originates from a switch in the polarity of the prevalent charge species. The left side (p-branch) reflects the increasing holes density, while the right one (n-branch) accounts for negative charge carriers (electrons). The density of charge carriers, and consequently the transistor current, reaches a minimum at the Dirac point (or charge neutrality point) in between the two branches, where the populations of holes and e^−^ are equal. During sensing experiments, as the biorecognition event takes place, the surface charge varies inducing a shift of the Dirac point. The most common approach to transduce the signal exploits the dependency of the Dirac point voltage on the analyte concentration, while other detection methods include the use of transconductance or I_d_ at fixed gate and drain potentials [179].

**Fig. 5:**
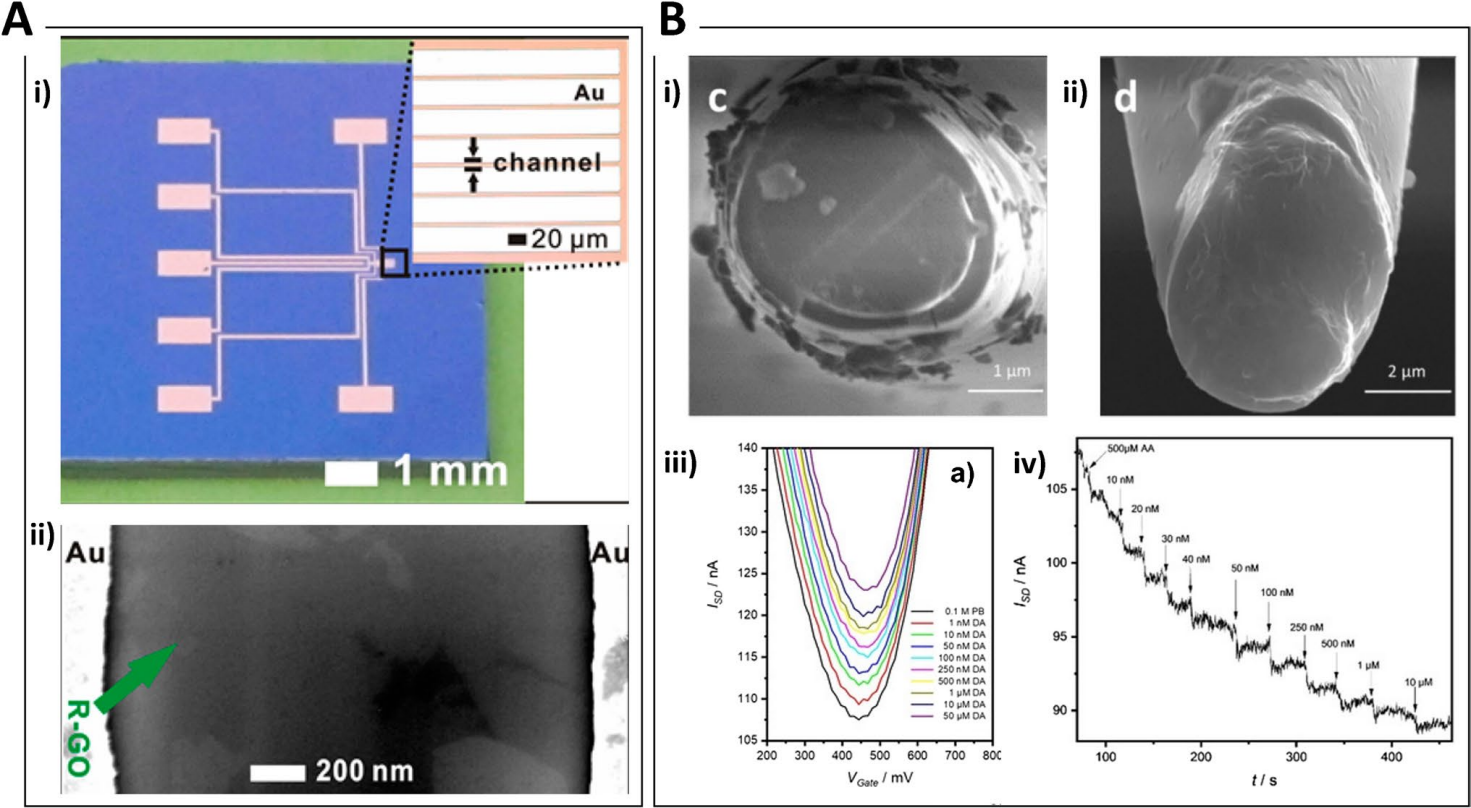
**A** r-GO FET biosensor chip. i) Optical images of the r-GO FET (inset: six individual sensors array); ii) SEM image of a single r-GO sheet spanning across Au electrodes. Reprinted with permission from [187]. Copyright 2014 American Chemical Society. **B** Spearhead-type GFET. i) Double barrel carbon nanoelectrodes after the polishing process, and ii) after the electrochemical pulse deposition of GO; iii) transfer curves recorded in 0.1 M PBS pH 7.4 with increasing DA concentrations at the graphene-oxide-based needle-type FET; iv) chronoamperometric measurement of I_d_ at V_g_ =  + 600 mV and V_d_ =  + 50 mV upon initial addition of AA followed by the addition of increasing concentrations of DA. Reprinted with permission from [174]. Copyright 2020 WILEY–VCH

The main fabrication procedures of graphene include the Scotch tape method [181], chemical vapor deposition [182], and deposition of graphene oxide followed by a reduction process [183]. Since the latter two methods are compatible with established microfabrication processes, an increasing number of articles dealing with micrometric and nanometric GFET sensors have been published in recent years. Among micrometric GFETs, the main detection strategy involves the chemical modification of the graphene channel with bio-recognizing elements, such as DNA [184–186], peptide nucleic acid (PNA, see, for instance, Fig. 5A) [187–189], proteins [190–193], and antibodies [194–200], which are able to specifically bind the target molecules. When the recognition element binds the target molecule, the surface charge or its distribution is modified upon variation of the Dirac point. For instance, negatively charged molecules induce an n-doping effect on the GFET sensor, moving the Dirac point toward negative values [200]. The high performances of these devices are demonstrated by the very low LODs that are usually in the range of fM [184, 185, 187, 188, 200, 201]. A micro-sized GFET for HIV identification has been fabricated by modifying the transistor channel with the antibody p-24, exhibiting a LOD of 100 pg/mL [198]. COVID-19 detection has been carried out down to 0.37 fM upon recognition of its RNA strand binding to the complementary RNA chain immobilized on the channel surface [201]. The determination of specific DNA or RNA molecules is accomplished following the interaction with the nucleic acid (PNA or DNA) chain containing the complementary basis sequence and typical LOD values are in the range 10–100 fM [184, 185, 187–189]. The use of nucleic acids overcomes the simple detection of the complementary chains, thanks to the choice of aptamers, which have been exploited to sense cytokines [202, 203], ochratoxin A [204], thrombin [205], and 17β-estradiol [206] with LOD values around 1 pM. The immunosensing approach has been exploited for the detection of specific biomarkers for Alzheimer [195, 200], cancer [196, 197], heart failure [199], cardiovascular disorders [198], and venous thrombosis [194]. A micro-sized GFET sensor for Hg has been reported that exploits the modification of the transistor channel with gold nanoparticles functionalized with thioglycolic acid to reach a LOD value of 25 nM, caused by a chelation reaction between the metal ion and the carboxylic groups [207]. Lastly, a GFET has been employed as pH sensor, following the consolidated approach employed for ISFETs [208]. It is worthy to note that Zhang’s group has devoted great research efforts to studying micro-sized GFET sensors, by developing and designing a micrometric GFET architecture characterized by a channel length of 4 µm, in a structure of interdigitated electrodes with a whole area of 200 × 200 µm^2^. Several of the above-mentioned devices have been realized with this technology, demonstrating the reliability of proposed platform [187–190, 199, 201, 209]. Most of the authors have highlighted the possibility to produce lab-on-a-chip sensing arrays for point-of-care applications related to the early diagnosis as the main advantage associated with the realization of micrometer-sized GFET sensors, due to the selective identification of specific biomarkers. Xu et al. [184] addressed the issues due to the different modifications that must be performed in GFETs placed on the same chip. An array of 8 sensors was fabricated, and a procedure has been developed for selectively anchoring two different DNA probes on different graphene channels.

Advanced applications of micro-sized GFETs deal with the monitoring of biological systems also at the single cell level, as well as the development of wearable sensors. Wang et al. [202] have proposed an ultra-flexible and stretchable GFET sensor able of conforming to underlying skin or tissue surfaces when they undergo large deformations. The devices were successfully placed on human skin and integrated into contact lenses. In a more recent paper, the same authors have demonstrated the use of this platform for the detection of cytokine in interferons IFN-γ and cancer biomarker in undiluted human sweat with a LOD of 740 fM [203]. Li et al. [190] have exploited a GFET modified with human metabotropic glutamate receptor 1 alpha for the monitoring of glutamate released from a primary culture of neurons, and the results suggested that these sensors could be useful for the study of the nervous system. Quast et al. [174] proposed a spearhead reduced graphene oxide (rGO)–based FET as dopamine sensor with boosted performance with respect to commonly used electrochemical methods (Fig. 5B). The device can operate in very small volumes and can be precisely positioned at the desired measurement location, thus providing enhanced spatial resolution during single cells recordings. NO detection has been accomplished by modifying the transistor channel with porphyrin [209] or hemin [210] through π–π stacking with the graphene layer. Both devices respond rapidly to NO in physiological environments with a sub-nanomolar sensitivity and have been exploited for monitoring the NO released by living cells, highlighting their use in complex biological systems. Kumar et al. [211] have developed a GFET to identify the presence of antibiotic resistant bacteria, using suitable peptide probes immobilized on the transistor channel. The use of dielectrophoresis allowed to detect individual bacterial cells in 5 min.

Very recently, new architectures have been developed to explore innovative transduction strategies and improve sensing performances. The realization of a nanopore on a micrometric channel made of graphene nanoribbons was exploited for the detection of DNA single molecules. In particular, the device was able to detect the passage of a double-stranded DNA through the nanopore, and the results have been validated by the measurement of an ionic current flowing through the nanopore [212]. Zhang et al. [194] have proposed a layer-by-layer assembled sandwich structure of rGO and rGO-encapsulated TiO_2_ to produce a biosensor for proteins detection. Since TiO_2_ promotes a photocatalytic process that renews GFET after the use, the device could be exploited for an increased number of determinations.

### OECT sensors

Organic electrochemical transistors (Scheme 1C) are a class of electrolyte-gated transistors where, due to the ion-permeability of the channel material, electrochemical reactions in the bulk of the semiconductor [213] rather than field effects at the interface modulate its conductivity. The organic material is typically PEDOT:PSS, where holes (PEDOT^+^) are responsible for electrical conduction in the conjugated backbone. The gate electrode, which can be either metal or polymer based, is in contact with the electrolyte solution and its potential (gate voltage, V_g_) drives ionic fluxes in solution and affects the channel conductivity. In fact, upon gating across the electrolyte, the doping state of the PEDOT channel can be reversibly modulated via ion-to-electron conversion phenomena assisting the redox reactions involved in holes extraction/injection [214, 215]. The change in bulk conductivity due to electrochemical doping processes involving the whole channel volume defines the unique electrical properties of these devices. In particular, large modulations of the channel current are obtained at low V_g_ (< 1 V), thus leading to large transconductance (g_m_) values in the mS range for micrometer-scale devices [216]. The physical behavior of the OECT working in depletion mode was first described by Bernards et al. [217] and was derived from the FET equations, where the 2D capacitance at the interface is replaced by a volumetric capacitance. The device behavior can be in fact modelled considering that its figure of merit, g_m_, scales with C* (the volumetric capacitance of the semiconductor material) and ${~}^{Wd}\!\left/ \!{~}_{L}\right.$ (where W, d and L are the channel width, thickness and length, respectively) [216, 218, 219], thus highlighting the importance of the channel material and geometry in defining the efficiency of the ion-to-electron conversion.

Virtually any kind of chemical species can be detected at an OECT sensor, as long as it is involved in (electro)chemical reactions that affect the electrochemical doping processes ruling the transistor response. Overall, both potentiometric and amperometric signals can be amplified using the drain current, I_d_, thus often outperforming the analytical response of the simple electrochemical transducer [220–223]. In fact, thanks to the intrinsic signal amplification and high current gains achievable with the transistor configuration, remarkable sensing performances can be reached already with millimeter scale sensors. Millimeter- and micrometer-scale OECT-based chemical sensors have been developed exploiting either a library of consolidated electrochemical transducers or more innovative approaches. The most common strategy employed for sensor development consists in the functionalization of the gate electrode with a proper transducer, including carbon nanomaterials [224], noble metals [225], organic semiconductors [221, 226, 227], and their composites [228], layered double hydroxides [223], and biorecognition elements [229–232]. From an analytical point of view, the absence of a reference electrode is a hotspot that inescapably deprives the sensing architecture of a stable reference system and impacts on the reliability of the sensing scheme. However, this peculiarity, together with the advancements in functional materials research, has opened unprecedented opportunities in chemical sensing applications that would be hardly accessible to conventional electrochemical approaches, including wearable chemical sensing with flexible [233], textile [234], and paper-based platforms [235].

Miniaturization of OECT-based chemical sensors has been achieved through photolithographic and pulling techniques, leading to planar chips or needle-type configurations, respectively, where at least one of the two transistor elements (gate or channel) has a dimension below 25 μm. This has brought unique advantages in reaching maximized gains, especially low detection limits, as well as high spatial and temporal resolution when OECT-based microsensors have been applied to the detection of neurotransmitters, enzymatic sensing, and cell monitoring.

Recently, an example of chemical microsensor based on the OECT architecture has been reported for dopamine detection. The work by Ferro et al. [236] describes the design of a planar (p-OECT) and a rolled-up (r-OECT) OECT configuration, both obtained by standard photolithography and thin-film deposition processes, consisting of three Au terminals and a copper (II) phthalocyanine (CuPc) molecular film as the channel, having CuPc already been explored as p-type organic semiconductor in low-power OFETs [237]. The r-OECT was obtained after the etching of a GeO_2_ sacrificial layer, which caused the active region of the device to roll-up, resulting in a self-curling nanomembrane with the OECT located in the microcylindrical cavity’s inner walls (W = 57 μm and L = 20 μm). R-OECTs show better transistor performances when compared to the planar counterpart and surpass the state-of-the-art electrolyte-gated transistors with a record intrinsic gain (e.g., g_m_ normalized by the channel conductance and expressed as a function of ${~}^{Wd}\!\left/ \!{~}_{L}\right.$) > 10^4^. Such a high figure of merit was attributed to the more efficient ion transport due to the intensification of the electric field in the confined microtubular environment. The potential of the rOECT for sensing applications was proven with the detection of DA in a pL volume of buffer solution filling the tubular cavity. The charge transfer between CuPc and DA during its electrooxidation at the transistor channel caused I_d_ to decrease and the ratio I_on_/I_off_ from the I_d_-V_g_ curves recorded at different DA concentrations was chosen as the analytical signal. Despite the impressive figure of merits, when used a chemical sensor the r-OECT reached a 3 µM LOD that is only relevant in urine analysis.

The development of low-cost point-of-care biosensing platforms for the simultaneous detection of multiple analytes with high sensitivity and selectivity poses several challenges to conventional electrochemical approaches. A multiplexed microfluidic platform for glucose, lactate, and cholesterol detection in human saliva was developed integrating a microarray of OECT biosensors [238]. The device architecture consisted of four OECTs with a PEDOT:PSS channel having W/L of 100/10 (μm/μm) and a planar Au/PEDOT:PSS gate of 500 × 500 μm^2^, which was modified by drop-casting of a mixture containing the enzyme (GOx, lactate oxidase (LOx), or cholesterol oxidase (ChOx)) or BSA (control, for background signal correction), chitosan and a redox mediator. The platform was integrated with a pumpless (finger-powered) poly(dimethylsiloxane) (PDMS)-based microfluidic allowing the saliva sample to flow across the sensing microarray. Elimination of electrical crosstalk was achieved upon isolation of the individual grounds (the source electrodes), while no chemical interference was observed thanks to the selectivity guaranteed by the enzymatic functionalization. Alternative to p-doping, only few cases of electron-transporting (n-type) organic semiconductors have been recently proposed for the fabrication of OECTs operating in accumulation mode. The use of n-type-doped conjugated polymers in OECTs is challenging due to (i) the well-known instability of carbanions that are easily oxidized in contact with air or water [239], and (ii) the low-voltage operation and high transconductance required for bioelectronics applications. However, accumulation-mode OECTs, where the channel is normally in the OFF state until electrochemically doped, holds promise towards the design of less power consuming devices. Pappa et al. have reported a micrometer-scale OECT (channel W and L equal to 100 and 10 μm, respectively) for lactate sensing, employing a n-type polymer as gate and channel material [240]. The so-called P-90 is a copolymer including a highly electron-deficient naphthalene 1,4,5,8-tetracarboxylic diimide repeat unit and an electron rich unsubstituted bithiophene repeat unit. Moreover, polar glycol side chains were alternated to nonpolar branched alkyl groups to enhance water uptake and ion transport and to facilitate enzyme conjugation. Following LOx drop-casting on the whole active area (channel and gate), lactate oxidation was induced at the channel where the n-type material could accept electrons from the enzymatic reaction in the absence of an additional redox mediator. Although the sensing performances were comparable to amperometric biosensors, still the simple OECT architecture eliminates the need of a reference electrode and is appealing for all those emerging applications where conventional electrochemical setups are less convenient, such as portable electronics.

**Fig. 6:**
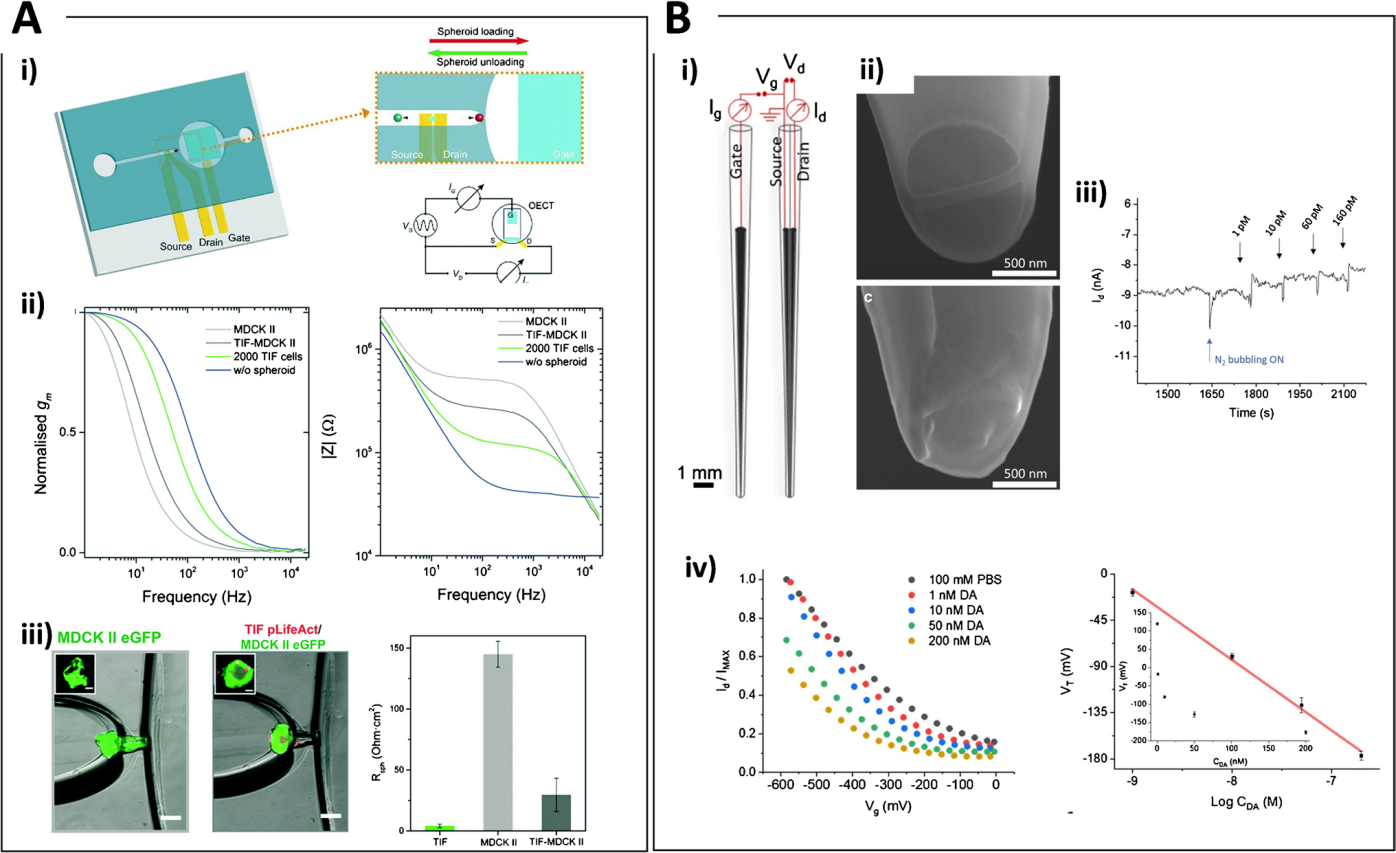
**A** Planar impedance sensor for 3D spheroids. i) Schematic representation of the microtrap impedance sensing platform. An OECT was fabricated on glass and the PDMS microtrapping device was positioned on top of the planar electronics. The transistor channel (25 × 25 μm^2^) is located to the left of the nozzle trap while the gate is positioned right after the microtrap. Zoom in illustration shows the loading (red) and unloading (green) flow direction to place the spheroid within the microtrap. Below, the wiring diagram of an OECT for cell impedance-based sensing is reported. ii) Typical frequency dependent response of the OECT in the absence (blue) and in the presence of TIF pLifeACT spheroid (green), TIF pLifeAct/MDCK II eGFP co-cultured spheroid (grey) and MDCK II eGFP spheroid (light grey). iii) From left to right: MDCK II eGFP spheroid (Ø = ca. 145 μm) trapped inside the circular-shaped nozzle (scale bar 100 μm, inset 50 μm); TIF pLifeAct/MDCK II eGFP co-cultured spheroid (Ø = ca. 183 μm) trapped inside the circular-shaped nozzle (scale bar 100 μm, inset 50 μm); typical impedance spectra in the absence (blue) and in the presence of TIF pLifeACT spheroid (green), TIF pLifeAct/MDCK II eGFP co-cultured spheroid (gray) and MDCK II eGFP spheroid (light grey). Reproduced from [242] with permission from the Royal Society of Chemistry. **B** Needle-type OECT for spatially resolved detection of dopamine. i) Configuration of the needle-type OECT. ii) SEM pictures of the dbCNE before and after PEDOT:PSS pulse electrodeposition to obtain the transistor channel bridging the two barrels. iii) I_d_ vs. time curve recorded during additions of DA in the range from 1 to 160 pM. iv) (left) Transfer curves recorded in presence of increasing amounts of DA. (right) Threshold voltage response to the concentration (inset) and logarithm of DA concentration in the nM range. Reproduced with permission from [173]. Copyright 2020 Springer Nature

Thanks to the low-voltage operation, stability in aqueous environment, and mixed conduction, micrometer-sized OECTs have been successfully applied to cell monitoring. The versatility of the electrolyte-gated transistor architecture enables different sensing strategies to be pursued. In a recent paper, an array of PEDOT:PSS channels (500 × 20 μm^2^) equipped with Pt-wire gate electrodes in cell culture medium was employed to detect the senescent green vegetative phase of *Haematococcus pluvialis* cells, e.g., the optimal induction stage for the highest production of a specific anti-oxidant of interest for commercial fermenters [241]. Due to the exposure to blue light and NaHCO_3_, the induced settlement of cells on the polymer channel was monitored in real-time via I_d_ recording, allowing to discriminate the onset and maturation stages of the senescent green maturation phase. The increase in I_d_ during cells settlement, which was proportional to cells density, was attributed by the authors to the change in the potential at the channel/cells interface due to the negative Z potential of *H. pluvialis* cells that directly induced the modulation of the channel current of the OECT. In another contribution, a PEDOT:PSS-based OECT was combined with a microfluidic trapping platform to accurately measure the impedance of 3D spheroids for drug screening applications [242] (Fig. 6A). In order to allow the integration of PDMS-based microfluidics and improve the adhesion between PEDOT and the gold pads, the authors developed a new photolithographic fabrication protocol based on self-assembled monolayer (SAM) formation. The microfluidic trap consisted of a PDMS single channel terminating with a nozzle trap geometry, with the function of blocking the spheroid between gate and channel, where its ion-permeability effectively modulates the OECT response. Upon acquisition of the transistor bandwidth spectra in the presence of mono- and co-cultured spheroids consisting of fibroblasts and epithelial cells, the authors showed marked differences in the spheroid resistance (R_sph_, attributed to the ionic paracellular resistance) depending on cells type and junctions. Moreover, the platform was validated quantitatively by measuring R_sph_ over time upon administration of a porogenic agent to the cell media. The advantages of micrometer-sized OECTs in terms of signal amplification and high-speed operation were exploited to monitor the extracellular electron transfer (EET) involved in the process of microbial respiration with electron transfer to extracellular acceptors [243]. *Shewanella oneidensis* MR-1 was attached by a chronoamperometric method onto a PEDOT:PSS/PVA gate electrode (500 × 500 µm^2^), which acted as an electron collector during the bacterial lactate metabolism under anaerobic conditions. Following the application of the optimized potentials (V_g_ =  + 0.3 V; V_d_ =  − 0.3 V), every electron transferred by the bacteria to the positively polarized gate pushed a cation from the electrolyte to enter the channel (100 × 10 µm^2^), thus leading to the extraction of a hole with the consequent de-doping (reduction) of PEDOT. The detection of the metabolic event taking place at the electrogenic bacteria occurred in the minute timescale. Although I_d_ reached saturation after ≈ 40 min from the addition of lactate with a relative change of only 1.6%, the amplification was highlighted by the absence of detectable changes in I_g_ upon lactate addition. Moreover, when probed using EIS in a two-electrode configuration, the small bacterial loading did not allow significant EET detection through the recording of temporal lactate consumption.

The fabrication of sub-micrometric organic channels is nowadays a challenging and exciting milestone towards the achievement of high operation speed and transistor density within bioelectronic interfaces, as well as high spatial resolution upon bioelectronic signals recording. These opportunities have been inspiring cutting-edge approaches that enable the further downscaling of the organic devices, including the design of vertically stacked contacts [244], electron-beam lithography [245], and electromigration-induced break junction on electrode gaps [246]. OECTs with nanometer-scale channels have been recently applied as neuromorphic devices to artificial synapses [247, 248]. Focusing on sensing applications, the only example of a sub-micrometric OECT (300 nm and 900 nm diameter channel and gate, respectively) published to date was obtained in a needle-type configuration [173], whose high aspect ratio allows precise positioning by means of a macroscopic handle and whose size is compatible with single-cell analysis (Fig. 6B). The transistor gate and channel were obtained upon PEDOT:PSS pulse electrodeposition on single- and double-barrel CNEs, respectively. The ability of the spearhead OECT to amplify amperometric signals was demonstrated for DA detection, exhibiting a threshold voltage variation of 69 mV decade^−1^ and achieving a detection limit of 1 pM during potentiostatic measurements of I_d_ in buffer solution.

## Conclusion and perspectives

The perspective of downscaling electrochemical probes is an essential technological evolution in the field of electrochemical sensing and its great potential towards the disclosure and transduction of transient, highly localized, and multiple chemical information in real time, which are inaccessible to any other sensing technique, has fascinated researchers since long time. In this regard, miniaturized electrochemical probes offer unique advantages in terms of reaching especially low detection limits and high resolution, in addition to the facile integration with other components and complementary techniques. Although outstanding progress has been achieved so far in the fabrication, modelling, and application of micro- and nanoelectrodes in the most important fields of electroanalysis, several challenges that limit most works at the proof-of-concept stage still need to be addressed. First, a key target is the achievement of a reproducible fabrication of the miniaturized probe, which clearly relates to the fabrication procedure. Due to the more consolidated techniques established for micropipettes and microchips, electrochemical probes in the micro-domain are nowadays commercially available in the interdigitated array, transistor, and pipette configurations. In contrast, the achievement of an accurate geometry control at the nano scale remains highly challenging due to (i) the ultra-high resolution required for nano-patterning and (ii) the difficulty in consistently characterize the nanometric probe. Concerning nanometric chip-like geometries, promising results have been achieved using electron-beam and nanoimprinting lithography [249], while for nanopipette-like structures, consistent advancements in terms of reproducibility and potential automatization have been recently reported in the fabrication of pyrolyzed carbon nanoelectrodes [250]. However, deviations from the expected electrochemical behavior at the nanoscale have demonstrated the inadequacy of theory predictions based on the steady-state current for the estimation of the UME dimensions, thus highlighting the inescapable need of using electron imaging techniques to characterize the actual nanoelectrode look [15, 250]. Another challenge relates to the validation of the analytical data. The consistency of the information accessed by micro- and nanometric electrochemical sensors is difficult to evaluate due to the lack of independent and comparably powerful analytical methods. The availability of alternative sensing techniques would also be useful, for instance, to understand the impact of the possible inflammation and physiological perturbation induced by micro- and nano-electrochemical sensors during in vivo recordings. Lastly, the miniaturization of the whole electrochemical setup and not only of the sensing probe (working electrode/indicator/transistor channel or gate) is evidently one of the most demanding challenges that micro/nano-sensors have been experiencing. This particularly concerns, for example, the removal of liquid components and has dramatic consequences on reference electrodes in view of their integration and miniaturization. A potential drift resulting from a parasitic non-zero current of 1 pA only (leading to a current density of ca. 130 μA cm^−2^ in case of a 1-μm-diameter disk microsensor) is incompatible with the measurement of an OCP [56]. While solid-state electrodes have been thoroughly discussed, micro-devices with an internal reference (such as a ferrocene-modified CF surface) could be further investigated [251] and innovative sensing systems where the potentiometric signal is exploited in combination with a reference-free transistor structure have recently been reported [222, 228, 252]. The transistor architecture offers in fact several advantages in terms of miniaturization opportunities. Although the low technological maturity, electrolyte-gated devices are proving impressive versatility in a set of bioelectronic applications where not only conventional electrochemical sensors, but also silicon-based electronics are inadequate because mechanical flexibility, low-power consumption, low-cost heterogeneous integration of electronics, light generation or detection, or bioprocessing are needed [253].
